# SwitchTFI: identifying transcription factors driving cell differentiation

**DOI:** 10.1186/s13059-025-03876-0

**Published:** 2025-12-02

**Authors:** Paul Martini, Anne Hartebrodt, Gustavo P. de Almeida, Carl-Philipp Hackstein, Dietmar Zehn, David B. Blumenthal

**Affiliations:** 1 Biomedical Network Science Lab, Department Artificial Intelligence in Biomedical Engineering, Friedrich-Alexander-Universität Erlangen-Nürnberg, Nürnberger Str. 74, Erlangen, 91052 Germany; 2 Division of Animal Physiology and Immunology, TUM School of Life Sciences and Center for Infection Prevention (ZIP), Technical University of Munich, Liesel-Beckmann-Str. 1, Freising, 85354 Germany; 3 Institute for Molecular Immunology, TUM School of Life Sciences and Center for Infection Prevention (ZIP), Technical University of Munich, Liesel-Beckmann-Str. 1, Freising, 85354 Germany

**Keywords:** Cell differentiation, Gene regulatory networks, Transcription factors, Single-cell gene expression data

## Abstract

Many methods exist that infer cell differentiation trajectories from single-cell RNA sequencing data, but only few determine which mechanisms drive the inferred differentiation dynamics. To close this gap, we developed the algorithm and Python package SwitchTFI. Utilizing regression stump learning, permutation-based family-wise error rate control, and node centrality measures, SwitchTFI identifies differentiation-driving gene regulatory networks and the key transcription factors involved in them. Comprehensive tests on pancreatic endocrinogenesis, erythrocyte differentiation, and T cell exhaustion datasets show that SwitchTFI can rediscover known differentiation factors, that it can discover novel biologically plausible hypotheses, and that it compares favorably to competitor methods.

**Supplementary Information:**

The online version contains supplementary material available at 10.1186/s13059-025-03876-0.

## Background

The process of cell differentiation is mirrored in the transcriptional landscape [1], where cells navigate by committing to lineage trajectories, leading to their distinct cellular identity and function. Single-cell RNA sequencing (scRNA-seq) provides a detailed view at the transcriptomic profile of individual cells at different development stages and is thus a great tool for elucidating cell differentiation. However, scRNA-seq data is usually captured at a single time point, making the reconstructing of the developmental trajectories an important problem in data-driven systems biology. While numerous methods for computational trajectory inference exist [2–6], relatively little work has been done in developing methods that determine the biological mechanisms and key transcription factors (TFs) that drive cell differentiation along an identified trajectory. Notable exceptions are the tools CellRank [6] (an improved version of Palantir [5]), spliceJAC [7], and DrivAER [8]. However, all of these tools are subject to important limitations, as summarized in the following paragraphs (see Additional file 1: Section 1 for further details).

Palantir identifies branches of the developmental trajectory by analyzing the properties of a Markov process on a nearest-neighbour graph embedding of the scRNA-seq data. This yields a probability distribution over the identified branches for each cell. The Pearson correlation between the expression of individual genes and branch probabilities is used to identify driver genes. CellRank improves upon Palantir by integrating RNA velocity [9, 10] for inferring the directionality of the edges in the graph embedding. Both methods suffer from the risk of encountering spurious correlations, since no functionality is provided to test the obtained correlations for statistical significance.

The spliceJAC method assumes a scRNA-seq count matrix with spliced and unspliced counts and a cell annotation vector with the respective cell states as an input to infer cell state-specific gene regulatory networks (GRNs), using a dynamical system model. Genes that are critical to the transition between cell states are found by analyzing the eigenspace of the Jacobian matrix associated with the differential equations of the starting cell state. By construction, the number of considered genes must be limited to the number of cells within a given cell state, such that an extensive preselection is required. In the tutorial published with the spliceJAC Python package, only 50 out of 27998 possible genes are used for GRN inference and transition driver gene identification. Notably, spliceJAC also provides a transition-specific GRN. However, it is constructed *post ho*c, using previously inferred marker and driver genes as vertices and the interactions between them as edges. Since no transition-specific information is utilized for predicting these interactions, their relevance to the cell state transition is unclear. Further, it is unclear how a sensible cutoff for the vertices and edges to be included in the GRN should be defined.

Given a scRNA-seq count matrix, a family of gene sets, and a phenotype vector of interest, DrivAER combines a deep count autoencoder network [11] for denoising and imputation of scRNA-seq data with a random forest model to compute relevance scores for the individual gene sets. TFs that drive differentiation can be found by defining the family of gene sets as a collection of sets of target genes of known TFs and using cell state annotations as the phenotype vector. Thus, mechanistic information is directly utilized for driver TF identification. However, since DrivAER works on the scale of TFs and their corresponding sets of target genes, it cannot recover which individual links between TFs and target genes are relevant.

To overcome the limitations of existing methods, we present SwitchTFI (**switch** **t**ranscription **f**actor **i**dentification) (see Table 1 for a high-level feature comparison against existing methods). SwitchTFI leverages the combined information of a scRNA-seq dataset annotated with progenitor and offspring cell states and a baseline GRN from an appropriate biological context to compute a transition GRN as an interpretable model of the regulatory mechanisms that drive cell state transitions. To infer the transition GRN from the baseline GRN, we define cell state transition relevance weights for all edges in the baseline GRN and then use a variant of the Westfall-Young permutation method [12] for family-wise error rate (FWER) correction to only keep those edges where these weights are significant. Subsequently, we use node centrality measures to identify key TFs within the transition GRN.

**Table 1:** Features of SwitchTFI and other methods for identifying differentiation driver genes

Method	CellRank	spliceJAC	DrivAER	SwitchTFI
Publication	[6]	[7]	[8]	This article
Input	scRNA-seq count matrix with spliced and unspliced counts or with pseudotime annotations	scRNA-seq count matrix with spliced and unspliced counts and with cell state annotations	scRNA-seq count matrix with cell state annotations and baseline GRN	scRNAseq count matrix with cell state annotations and baseline GRN
Output	Ranked genes	Ranked genes and transition GRN	Ranked TFs	Ranked TFs and transition GRN
Unit of analysis	Genes	Genes	TFs	Edges between TFs and target genes
Inbuilt statistical validation	None	None	None	Edge-level *P*-values

## Results

### Inferring transition GRNs with SwitchTFI

**Fig. 1:**
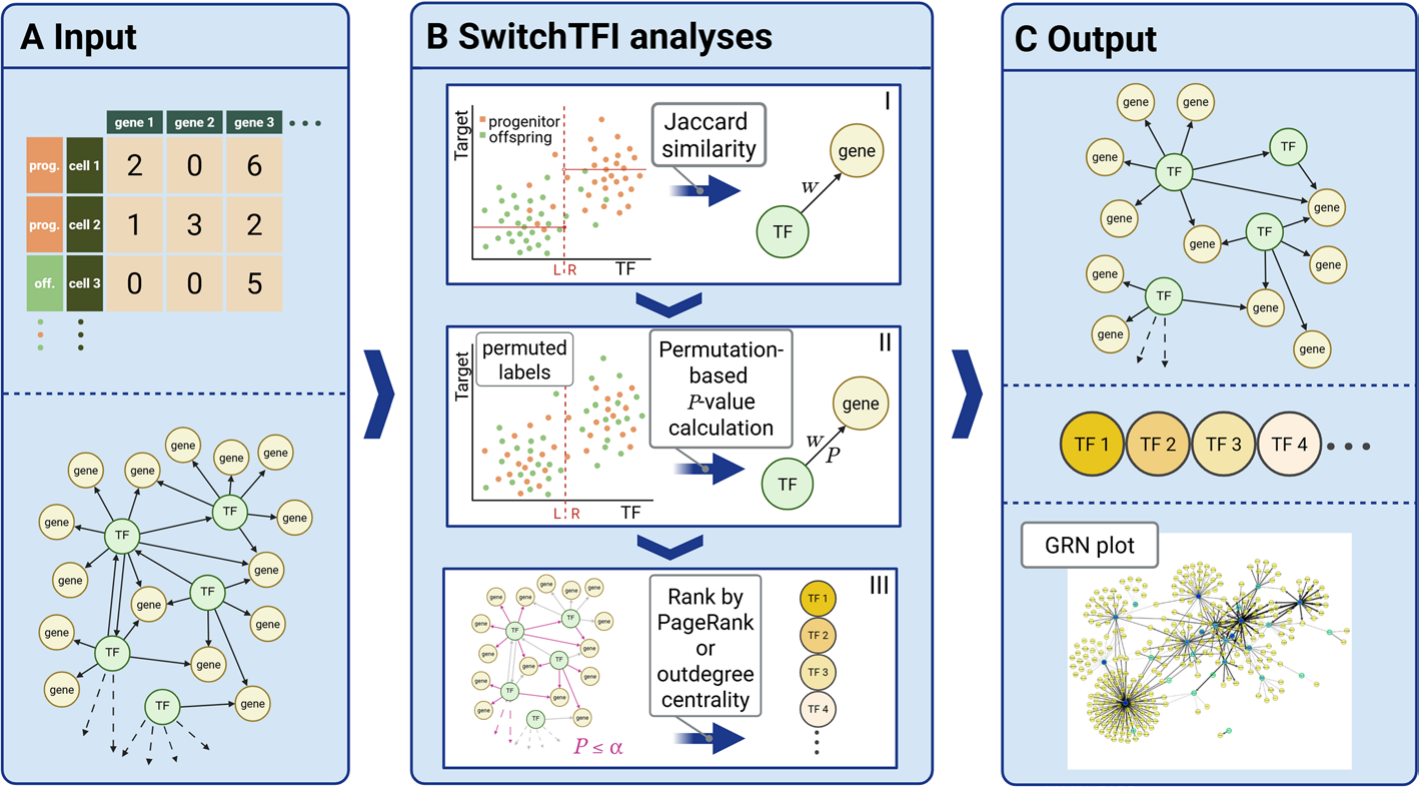
Overview of SwitchTFI. (**A**) Input: a scRNA-seq count matrix with progenitor/offspring annotations and GRN from a fitting biological context (possibly inferred from the scRNA-seq data). (**B**) Workflow: (I) weight fitting on MAGIC-imputed scRNA-seq data, (II) empirical P-value calculation, and (III) pruning of the GRN and ranking of TFs according to centrality in the transition GRN. (**C**) Output: transition GRN, ranked list of driver TFs, and plot of the transition GRN. Created in BioRender. Blumenthal D [85] https://BioRender.com/lka3ptt

An overview of the SwitchTFI method is provided in Fig. 1 (see “Methods” for details). As input, SwitchTFI requires scRNA-seq data from cells of annotated consecutive cell states (hereafter referred to as “progenitor” and “offspring” cells) and a baseline GRN containing the edges from TFs to target genes. For this study, we used Scenic [13] to infer the baseline GRN from the scRNA-seq data. Alternatively, expert-curated TF-target gene links obtained from databases such as CollecTRI [14] could be used as baseline GRN. For each edge *e* of the baseline GRN, we predict the target gene expression from the expression of the TF, using a regression tree of depth one and MAGIC imputation [15] to mitigate the problem of sparse TF-target gene co-expression. The regression tree’s decision boundary induces a partition of the cells. We compare this partition to the progenitor-offspring partition provided by the cell state annotations and obtain an edge weight $w_e\in [0.25,1]$ that quantifies similarity between the two partitions. SwitchTFI’s key idea is that the two partitions are similar if the TF-target gene link is relevant to the cell state transition (i.e., if the TF drives differentiation). In this case, the cell state annotations induce a distinct clustering of the cells in TF-target gene expression space, leading to a large edge weight $w_e$.

By adopting this edge-wise approach instead of analyzing single genes, SwitchTFI focuses on the smallest regulatory unit that still carries mechanistic information, i.e., TF-target gene links. To capture the regulatory mechanisms that drive differentiation on the scale of the GRN, SwitchTFI prunes edges from the baseline GRN, retaining only the ones most relevant to the cell state transition in a transition GRN. For this, we permute the cell state annotations, compute edge weights $\tilde{w}_e$ for the permuted cell states, and then compute adjusted Westfall-Young *P*-values $P_e$ to assess if $w_e$ is significantly larger than expected by chance. By retaining only edges with $P_e\le \alpha$, we obtain a transition GRN that is specific to the respective cell state transition with controlled $\text {FWER}\le \alpha$.

The transition GRN is a highly interpretable output format that is well suited for further computational and biological analyses. It can be visualized with the convenient plotting functionality provided by the Python implementation of SwitchTFI. Similar to existing methods, SwitchTFI also returns a ranked list of putative key driver TFs. SwitchTFI provides two methods to compute this ranked list: PageRank centrality [16], which is widely used for gene ranking in computational systems biology [17, 18], and weighted outdegree centrality as a conceptually simpler and more interpretable alternative. With both centrality measures, the TF ranking is mechanistically informed, as its computation is based on importance of the TFs within the transition GRN: TFs appear at the top of the ranked lists if they have many targets in the transition GRN (in the case of PageRank: many important targets), i. e., if SwitchTFI predicts them to be key regulators of the cell state transition from progenitor to offspring cells.

For the sake of a concise presentation, we used PageRank centrality for our in-depth analyses reported in the following two subsections. For the comparison against existing methods reported in the last subsections of the “Results”, we tested both PageRank and weighted outdegree centrality. The choice of the FWER threshold $\alpha$ mainly influences the size of the transition GRN, while the ranking of the TFs remains largely unchanged (see Additional file 1: Section 3). For all results reported in this article, we chose $\alpha = 0.05$, which results in a concise and well-interpretable output.

### Driver TFs identified by SwitchTFI and corresponding target genes show characteristic dynamics over pseudotime

To validate SwitchTFI and examine the validity of its underlying assumptions, we ran it on a scRNA-seq dataset of murine pancreatic tissue from embryonic day 15.5 by Bastidas-Ponce et al. [19, 20]. We used the cell type annotations provided with the dataset to subset pre-endocrine cells (progenitor cells) together with $\beta$- or $\alpha$-cells (offspring cells), resulting in two datasets: one representing $\beta$-cell development and the other $\alpha$-cell development. Moreover, we used the hematopoiesis dataset by Paul et al. [21, 22], which we subset to the erythrocyte lineage and annotated with progenitor–offspring labels by merging the fine-grained annotations provided with the dataset. We selected these datasets because pancreatic endocrinogenesis and haematopoiesis are well-studied processes that are often used as templates for developing and testing computational methods that aim at elucidating cellular differentiation [6, 7, 10, 23]. See “Methods” and “Availability of data and materials” for details on the datasets and their availability.

**Fig. 2:**
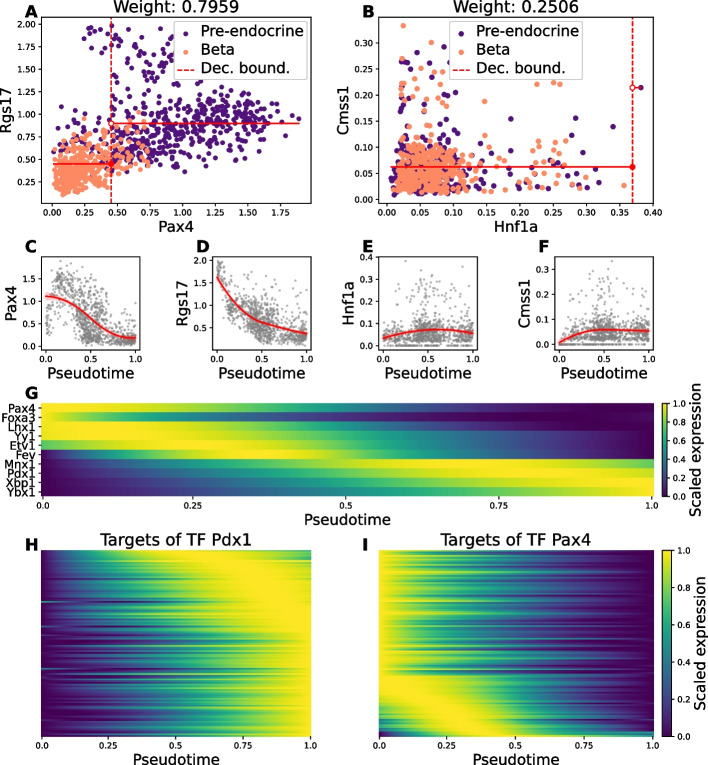
Gene expression (with MAGIC imputation) of key TFs and target genes uncovered by SwitchTFI on the mouse β-cell development data. (**A**, **B**) Scatter plots of TF expression (x-axis) and target gene expression (y-axis) of the cells in the scRNA-seq dataset. (**A**) corresponds to a highly and (**B**) to a lowly weighted edge. The regression stump fit during the weight fitting step is plotted in red. (**C** – **F**) Gene expression trends over pseudotime for the individual TFs and target genes of the edges shown in (**A**) and (**B**). (**G**) Expression trends over pseudotime for the top-10 TFs predicted to be relevant to the cell state transition. TFs have been sorted according to their trend’s peak in pseudotime. (**H**, **I**) Expression trends over pseudotime for the targets regulated by putative driver TFs Pdx1 and Pax4

Figure 2 provides a visual overview of SwitchTFI’s results on the $\beta$-cell development dataset. For highly weighted edges, the cell state annotations have a pronounced cluster structure that corresponds well to the partition induced by the regression tree (Fig. 2A, Additional file 1: Fig. S1). The converse can be observed for lowly weighted edges (Fig. 2B). Further, we looked at the shape of gene expression trends in pseudotime. Genes with strongly decreasing or increasing expression in pseudotime are generally adjacent to edges with a high weight (Fig. 2A, C, D). Nearly constant expression levels along the pseudotemporal trajectory are associated with low weights (Fig. 2B, E, F). Genes with rapid expression changes during differentiation are of interest for further investigation, since such changes can be linked to governing differentiation and changes in cell function. For the top 10 putative driver TFs, a rapid, switch-like increase or decrease in expression can be observed (Fig. 2G). This behavior carries over to the targets of the top TFs. For instance, Pdx1 is a TF that exhibits a marked increase in expression over pseudotime. The same can be observed for its targets (Fig. 2H), indicating the importance of the Pdx1 regulatory complex for developed $\beta$-cells. Similarly, the diminishing expression of Pax4 over pseudotime coincides with the downregulation of its targets (Fig. 2I). Thus, we can associate Pax4 with inducing and regulating differentiation towards the $\beta$-cell fate but declining importance to mature $\beta$-cells. Overall, these results show that SwitchTFI’s underlying methodological assumption that highly weighted edges point towards regulatory links being involved in cell state transition is reasonable.

**Fig. 3:**
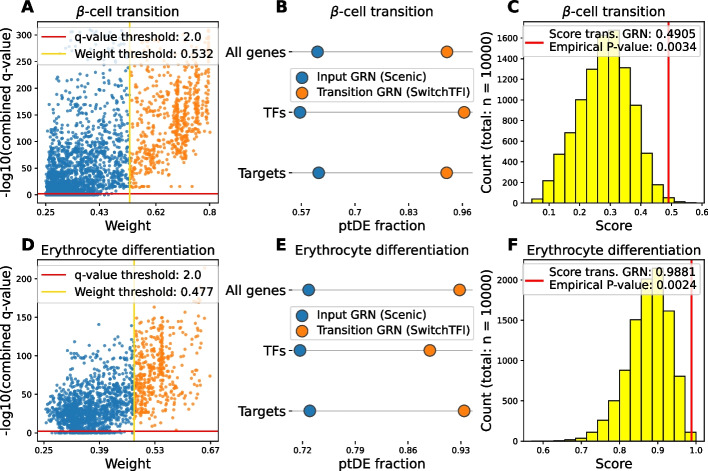
Quantitative inspection of SwitchTFI’s transition GRNs for the $\beta$-cell transition and the erythorcyte differentiation data (see Additional file 1: Fig. S2 for corresponding plots for the $\alpha$-cell transition dataset). (**A**, **D**) Scatter plot of edge weights vs. combined ptDE *q*-values of the unpruned input GRN computed by Scenic. Combined *q*-values are $-\log _{10}(\cdot )$-transformed. (**B**, **E)** Fraction of ptDE genes, TFs and targets in the Scenic and SwitchTFI GRN. (**C**, **F**) Histogram of the connectivity scores of randomly sampled subnetworks of the Scenic GRN. The transition GRN’s score is visualized as a red line

In addition to the qualitative validation shown in Fig. 2, we quantitatively examined SwitchTFI’s output with respect to the fraction of genes with biologically interesting, rapidly changing expression trends. To quantify a rapid change in gene expression, we tested for differential expression along a previously computed pseudotemporal trajectory (ptDE). A ptDE *q*-value was computed for each gene of the baseline GRN using switchde [24]. By pairwise combining the *q*-values of TFs and targets using Fisher’s method, we obtained edgewise ptDE *q*-values. Figure 3A and D visualize the edge weights and the combined log-transformed *q*-values for all edges of the unpruned baseline GRN computed by Scenic. Edges below the *q*-value threshold are deemed insignificant with respect to ptDE. Edges above the weight threshold have an adjusted permutation-based *P*-value below 0.05 and are thus retained in the transition GRN. Notably, all such edges have a significant combined *q*-value ($\le 0.01$), and we observe a clear correlation between large weights and small combined *q*-values (Pearson correlation coefficient between non log-transformed *q*-values and weights for $\beta$-cell transition: $-0.258$, erythrocytes: $-0.131$).

Figure 3A and D show that combined *q*-values are a relatively broad criterion for quantifying the relevance of an edge in the baseline GRN to the cell state transition, as only few edges do not have a significant combined *q*-value. Although the SwitchTFI algorithm does not rely on the combined *q*-values, we observe that SwitchTFI *de facto* makes a mechanistically informed selection among the edges with a significant ptDE combined *q*-value. The biological relevance of this selection is confirmed by the observation that the fraction of ptDE genes, TFs, and target genes in the transition GRN is substantially increased compared to the baseline GRN (Fig. 3B, E).

Further, we compared the number and size of the connected components in SwitchTFI’s transition GRNs against randomly sampled subnetworks of the baseline GRN. As a score for the comparison, we used the sum over the squared ratios of connected component sizes to the total number of vertices in the subnetwork. This way, more tightly connected subnetworks in which many nodes are concentrated in few connected components receive a higher connectivity score (for details see Eq. 8). Figure 3C and F show that the transition GRNs consist of significantly fewer and larger connected components than expected under the random background model, indicating that SwitchTFI’s transition GRNs indeed comprise biologically relevant internally connected regulatory units.

### SwitchTFI recovers key TFs involved in pancreatic endocrinogenesis

**Fig. 4:**
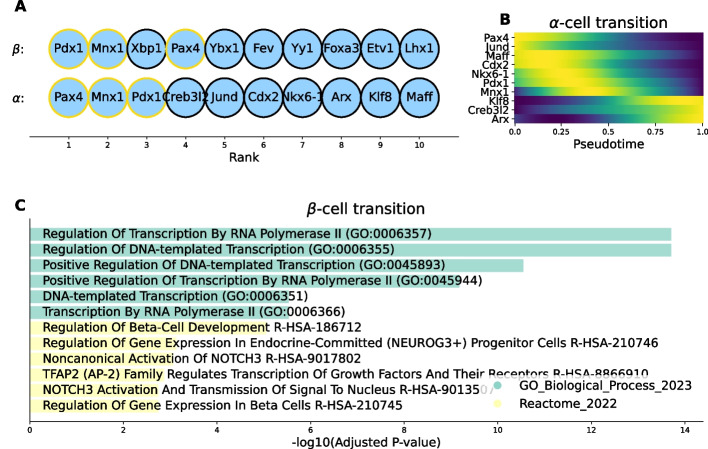
Qualitative validation of the top 10 pre-endocrine $\beta$-/$\alpha$-cell transition driver TFs, as ranked by SwitchTFI (see Additional file 1: Fig. S3 for corresponding results for the erythrocyte differentiation data, which are again in line with prior studies [27, 28]). **A** Rankings of the putative driver TFs. Yellow outline marks TFs appearing in both rankings. **B** Expression trends in pseudotime of the top 10 TFs predicted to drive the transition to $\alpha$-cells. TFs have been sorted according to their trends’ peaks in pseudotime. **C** Gene set enrichment results for the top 10 putative $\beta$ driver TFs. The top 6 terms of each reference database are ranked according to their adjusted *P*-value as computed by Enrichr

In Fig. 4, the top 10 pre-endocrine $\beta$- and $\alpha$-cell driver TFs as ranked by SwitchTFI are shown (Fig. 4A), together with the expression trends of the $\alpha$-cell drivers over pseudotime (Fig. 4B, see Fig. 2G above for the $\beta$-cell drivers’ expression trends over pseudotime). For both lists of driver TFs, we performed gene set enrichment analysis, using the Enrichr webtool [25, 26]. For the $\beta$-cell drivers, a significant enrichment can be observed for terms that relate to general regulation of transcription or more specifically regulation of $\beta$-cell development (Fig. 4C, see Additional file 1: Fig. S2 for enrichment results for $\alpha$-cell drivers), indicating that key TFs retrieved by SwitchTFI are functionally relevant. Moreover, for several of the top-ranked TFs, there is strong literature support for their involvement in pancreatic endocrinogenesis:

- Pdx1 appears among the top-ranked genes for the $\beta$- and $\alpha$-transition. This is unsurprising, since Pdx1 assumes a central role in governing endocrinogenesis [29–32] and its expression is essential to the development of the endocrine system [29]. Cellular reprogramming of $\beta$-cells to $\alpha$-like cells by Pdx1-deletion was experimentally confirmed [33]. Our results fully confirm this antithetical relevance of Pdx1 to differentiating $\beta$- and $\alpha$-cells (see contrary expression trends over pseudotime in Figs. 2G and 4B).
- The TF Xbp1 is highly ranked as a driver of the $\beta$-cell transition and shows an increasing expression over pseudotime (Fig. 2G). Xbp1 has been linked to differentiation processes in many cell types, including $\beta$-cells. In particular, it was shown that Xbp1 has a key role in maintaining $\beta$-cell identity and repressing transdifferentiation into other pancreatic islet cells, such as $\alpha$-cells [34].
- Arx is identified as key TF driving $\alpha$-cell development and exhibits an increasing expression over pseudotime (Fig. 4B). This is coherent with the finding that Arx expression is sufficient for maintaining $\alpha$-cell identity in the developing and neonatal pancreas [35, 36].
- Pax4 appears among the top 10 drivers inferred for both $\alpha$- and $\beta$-cell development and, for both trajectories, its expression peaks early in pseudotime (Figs. 2G, 4B). These findings are in line with experimental studies according to which Pax4 plays an important role in the regulation of pancreatic endocrine development [29, 37] but alone does not suffice to determine endocrine subtype fate [38].

**Fig. 5:**
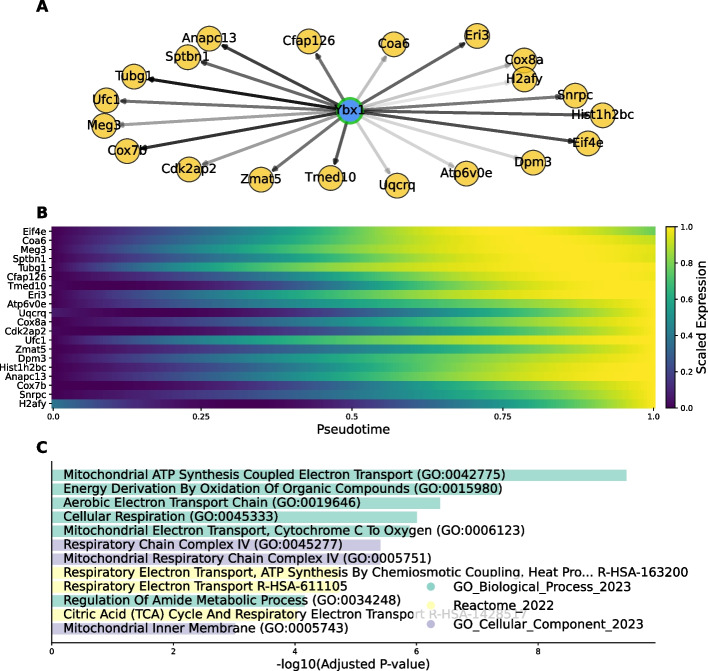
Exemplary hypothesis generation with SwitchTFI. **A** TF Ybx1 and its top 20 target genes, ranked according to SwitchTFI’s edge score computed from the edge weight and the empirical *P*-value (see Eq. 5). The shade of the edges is proportional to the scores, darker shades indicate higher relevance. The network plot was generated with SwitchTFI’s inbuilt plotting function switchtfi.plot_regulon(), which supports out-of-the-box visualization of inferred regulons. **B** Expression trends in pseudotime of the top 20 targets of Ybx1. **C** Gene set enrichment results for the top 20 targets of TF Ybx1. Terms are ranked according to their adjusted *P*-value as computed by Enrichr

Beyond these TFs which are known to be involved in pancreatic endocrinogenesis, SwitchTFI predicts Ybx1 as the fifth most important TF for $\beta$-cell development (Fig. 4A). Prior research has shown that Ybx1 is an important modulator of epithelial cell differentiation [39]. Ybx1 is also implicated in various disease pathways, where it appears to modulate epitelial-menenchymal transition [40–42], highlighting the potential implication of this gene in differentiation processes. Thus, we examined Ybx1’s top 20 predicted targets in the transition GRN (Fig. 5A). The expression values of these targets peak late in pseudotime (Fig. 5B), suggesting their involvement during the final stages of $\beta$-cell differentiation. Notably, two of them, Meg3 and Tmed10, have been linked to exocrine pancreatic differentiation in prior studies, especially in the context of diabetes mellitus [43–45]. Gene set enrichment analysis of the top 20 targets returned a collection of terms related to oxidative processes and energy metabolism (Fig. 5C). Oxidative stress due to reactive oxygen species (ROS) plays an important role in $\beta$-cell differentiation and maintenance [46]. ROS and mitochondrial deficiencies have also been linked to $\beta$-cell dysfunction and failure in type II diabetes (T2D) [47, 48]. Therefore, one could hypothesize that Ybx1 is implicated in the differentiation or maintenance of $\beta$-cells via the modulation of genes controlling the oxidative environment.

In sum, SwitchTFI is hence able to recover TFs that are biologically validated to drive differentiation towards known endocrine cell fates. Moreover, the example of the Ybx1 regulon showcases SwitchTFI’s potential for data-driven hypothesis generation in settings where the user has prior evidence on genes (Meg3 and Tmed10 in our example) involved in a cell state transition of interest and aims to identify candidate upstream regulators (here: Ybx1) and their action mechanisms to be validated in follow-up wet lab experiments.

### Comparison of SwitchTFI to other methods

To compare the results of SwitchTFI to the previously discussed competitor methods, we ran each on the pre-endocrine $\beta$-cell transition data and on the erythrocyte differentiation data. For the corresponding results for the pre-endocrine $\alpha$-cell transition data, we refer to Additional file 1: Fig. S2. SwitchTFI was run in two modes: with TFs ranked according to PageRank and according to their weighted outdegree in the transition GRN. Since only spliceJAC has a GRN as part of its output, we resorted to comparing the top 20 putative driver genes. Since the erythrocyte data does not come with spliced and unspliced counts, analysis with spliceJAC was not possible and CellRank had to be run in a non-default mode that does not rely on RNA velocity.

**Fig. 6:**
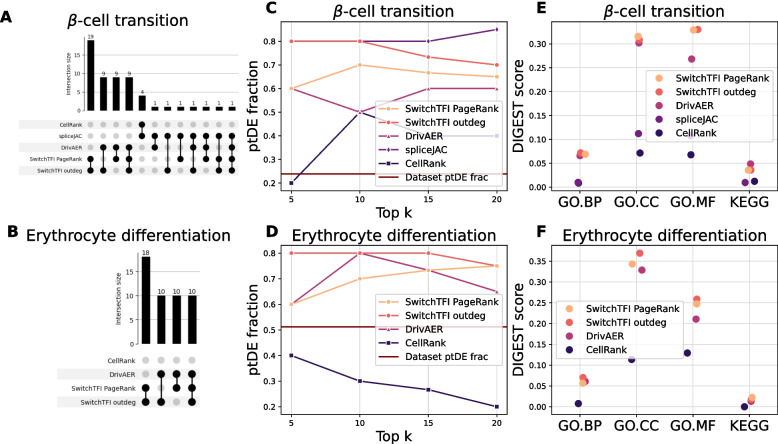
Comparison of SwitchTFI’s output to competitor methods. (**A**, **B**) UpSet plots visualizing the cardinality of the intersections of the top 20 putative driver gene sets. Empty intersections are not shown. (**C**, **D**) Fractions of ptDE genes among the top $k = 5, 10, 15, 20$ putative driver genes from each method. Higher is better. (**E**, **F**) Functional coherence scores for the sets of top 20 putative driver genes computed with DIGEST. Higher is better

Figure 6A and B show the overlaps between the top 20 putative driver genes computed by the different methods on the two datasets. Overlaps between the two versions of SwitchTFI are large on both datasets, and permutation tests show that, although the transition GRNs contain only 27–28 TFs (Additional file 1: Tables S3–S5), the large overlaps are not mere consequences of small numbers of TFs in the transition GRNs (Additional file 1: Fig. S4). This indicates that the choice of the node centrality score has a limited effect on the results and that SwitchTFI is thus robust with respect to the choice of this subroutine. Among the other methods, DrivAER has the largest overlap with SwitchTFI, sharing around 50% of the top 20 drivers. For CellRank and spliceJAC, overlaps with SwitchTFI are very small or empty, indicating that SwitchTFI returns complementary results. We hypothesize that this may be due to dissimilarities in input and output specifications of SwitchTFI and DrivAER, on the one hand, and CellRank and spliceJAC, on the other hand (Table 1): Both SwitchTFI and DrivAER rely on a baseline GRN as input while CellRank and spliceJAC do not; and both SwitchTFI and DrivAER return only TFs as potential cell differentiation drivers while CellRank and spliceJAC also return putative driver genes that are no TFs.

Figure 6C and D show the fractions of ptDE genes in the top $k\in \{5,10,15,20\}$ driver genes predicted by each method. The fraction of ptDE genes within each dataset serves as a baseline and is shown as a horizontal line. On both datasets, SwitchTFI is among the best-performing methods in terms of ptDE fraction. CellRank improves only marginally over the baseline ptDE fraction and in some cases fails to exceed it. DrivAER performs on par or slightly worse than SwitchTFI. On the $\beta$-cell development dataset, spliceJAC slightly outperforms SwitchTFI with respect to ptDE fraction (for the erythrocyte differentiation dataset, spliceJAC could not be used due to the lack of spliced and unspliced counts).

We further compared the list of top 20 drivers computed by the different methods with respect to functional coherence based on KEGG and GO annotations, relying on functional coherence scores which we computed with DIGEST [49]. In Fig. 6E and F, the functional coherence scores are compared; the predicted driver genes and full output of DIGEST can be found in Additional file 1: Tables S7–S10 (including empirical *P*-values which show that all methods yield coherence scores which are significantly larger than expected under a random background model). Remarkably, spliceJAC achieves rather low scores here despite performing well with respect to the ptDE-based metric. In contrast, SwitchTFI and DrivAER perform well, with SwitchTFI scoring the highest. Both directly utilize information on regulatory relationships between genes to compute driver genes.

Overall, these results lead to the conclusion that all of the compared methods can predict driver genes that display potentially relevant expression trends in pseudotime. However, the functional coherence of a proposed set of driver genes is substantially increased for methods which rely on mechanistic information, with SwitchTFI striking the best balance between ptDE fraction and functional coherence.

Finally, we compared runtime and memory requirements of the four tested methods, using data from a large-scale cell reprogramming experiment [50, 51] (Additional file 1: Fig. S5). For DrivAER and SwitchTFI, running Scenic to compute the baseline GRN required as input was the main bottleneck (this bottleneck could be resolved by replacing Scenic by a faster GRN inference method or by an expert-curated GRN such as CollecTRI [14]). Given all inputs, SwitchTFI showed the best scalability patterns of the four methods, both in terms of memory and runtime. For instance, when run on a comparatively large dataset with 10000 cells and a baseline GRN with 6435 edges, SwitchTFI terminated in around 30 minutes and required around 8 GB of main memory. These results show that SwitchTFI scales to realistic dataset sizes, especially because SwitchTFI should always be run on targeted progenitor/offspring populations and not on atlas-scale data covering a large variety of cell types.

### SwitchTFI identifies candidate regulators involved in T helper cell exhaustion

**Fig. 7:**
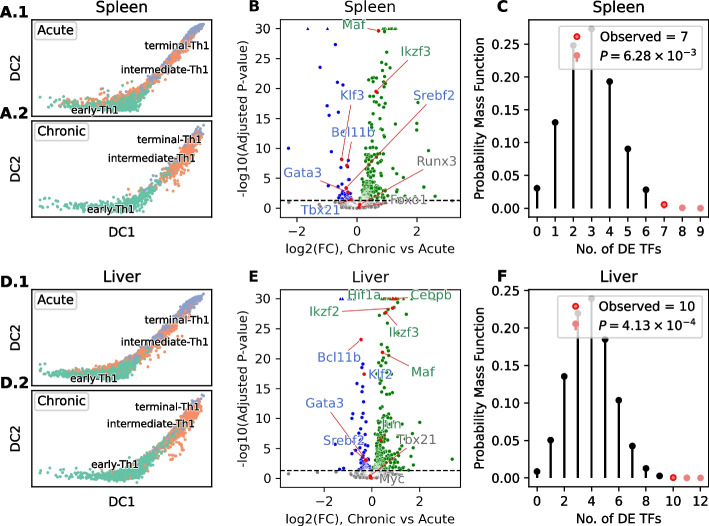
Results of T helper cell exhaustion case study. (**A**, **D**) First and second diffusion components (DC1 and DC2) of diffusion map embeddings of T cell scRNA-seq data from spleen (**A**) and liver (**D**) tissues of chronic and acute murine LCMV infection models. In the acute infection (A1, D1), Th1 cells reach a terminally differentiated state, whereas in chronic infection (A2, D2), differentiation stalls at an intermediate state. (**B**, **E**) Volcano plots showing results of differential expression analysis for all TFs in progenitor Th1 cells in the chronic and the acute infection in spleen (**B**) and liver (**E**), carried out with the ZINB-WaVE extension [56] of edgeR [57]. The horizontal dashed lines indicate an adjusted *P*-value threshold of 0.05. TFs that are significantly more strongly expressed in the chronic infection are marked in green; TFs that are significantly more strongly expressed in the acute infection are marked in blue. Driver TFs identified by SwitchTFI are highlighted in red. For better visualization, adjusted *P*-values below $10^{-30}$ were truncated and are indicated by triangular markers at the upper border of the plots. (**C**, **F**) Hypergeometric tests demonstrating the enrichment of driver TFs among differentially expressed TFs in spleen (**C**) and liver (**F**). The observed numbers of significant TFs (adjusted $P\le 0.05$) within the sets of driver TFs are indicated with red markers

To showcase how SwitchTFI can be used to derive novel and at the same biologically meaningful hypotheses for datasets where prior knowledge on cell state transition mechanisms is scarce, we used it to reanalyze scRNA-seq data [52] of CD4-positive type 1 T helper (Th1) cells from murine liver and spleen tissues 10 days post infection with the Armstrong and Docile strains of the lymphocytic choriomeningitis virus (LCMV) that induce an acute and a chronic infection, respectively [53, 54]. Diffusion maps computed with destiny [55] suggest that, in the acute infection, early Th1 cells develop through an intermediate state into terminally differentiated Th1 cells with effector capacities (Fig. 7A1, D1). In the chronic infection, cell development stops in the intermediate state (Fig. 7A2, D2), suggesting the existence of a Th1 exhaustion mechanism in the chronic infection that is linked to incomplete cell differentiation.

To identify driver TFs involved in this mechanism, we ran SwitchTFI on the data from the acute infection model (separately for each of the two tissues), using the early Th1 cells (green cells in Fig. 7A, D) as progenitors and the terminally differentiated Th1 cells (violet cells in Fig. 7A, D) as offspring cells. For both spleen and liver, we obtained very compact transition GRNs (Additional file 1: Figs. S6 and S7), containing nine (spleen) and twelve (liver) TFs predicted to be key factors in the transition towards the effector Th1 state in the acute infection. In both tissues, most of these TFs are differentially expressed between progenitor cells from the chronic and the acute infection (Fig. 7B, E) — significantly more than expected by chance given the overall fractions of differentially expressed TFs in the spleen and liver datasets (Fig. 7C, F). Moreover, six TFs appear in the intersection of the sets of driver TFs identified for the two tissues (Tbx21, Gata3, Bcl11b, Maf, Ikzf3, Srebf2), which is again significantly more than expected by chance ($P=2.15\times 10^{-11}$, hypergeometric test). Taken together, these results provide strong evidence that SwitchTFI’s transition GRNs pinpoint to cell differentiation mechanisms that are differentially active between the acute and the chronic infection and could thus explain the lack of terminally differentiated effector Th1 cells observed in the chronic infection model.

Beyond this quantitative evidence, the TFs identified by SwithTFI are also plausible from a biological point of view. Notably, SwitchTFI predicted TFs to be involved in the transition from progenitor to terminal Th1 cells in the acute infection that are essential for normal T helper differentiation and are moreover more strongly expressed in the progenitor cells from the acute infection. These TFs include Tbx21 (Fig. 7B), the key Th1 TF, Bcl11b (Fig. 7B, E), a TF repressing Th2 functionality [58–60], and Srbf2 (Fig. 7B, E), which regulates the metabolism of effector T cells [61]. Another interesting TF identified by SwitchTFI is Ikzf3, which encodes the zinc-finger protein Aiolos and is more strongly expressed in the progenitor cells from the chronic infection in both spleen and liver (Fig. 7B, E). Ikzf3 is among the top two ranked TFs predicted by SwitchTFI in both the liver and the spleen dataset and, in both datasets, exhibits a “switch on” behavior during the transition from progenitor to offspring cells (spleen: adjusted $P=1.66\times 10^{-20}$, liver: adjusted $P= 3.46\times 10^{-40}$, edgeR with ZINB-WaVE extension). Since prior studies have linked Aiolos inhibition to rescue from T cell exhaustion [62] and have found that Ikzf3 expression is negatively correlated with the formation of CD4-positive effector T cells and positively associated with the key exhaustion TF Tox [63], this suggests that absence of Ikzf3 in the progenitor population may be essential for proper T cell differentiation. Involvement of all of these predicted driver TFs in Th1 cell exhaustion could be tested experimentally utilizing existing transgenic mouse lines that allow the deletion or overexpression of the aforementioned TFs in Th1 cells.

## Discussion

We presented SwitchTFI, a GRN-based algorithm to identify transcription factors that drive cell differentiation from a progenitor to an offspring condition. We evaluated SwitchTFI on pancreatic endocrinogenesis and erythrocyte differentiation datasets and showed that it uncovers TFs known to be involved in these development processes. Moreover, SwitchTFI compares excellently against existing methods in terms of (i) functional coherence and (ii) fractions of predicted drivers that are differentially expressed over pseudotime. With respect to the latter metric, the best-performing competitor is spliceJAC, but unlike SwitchTFI, this method requires both spliced and unspliced counts as input, which are not always available. In terms of functional coherence, DrivAER is the first competitor, but is clearly outperformed by SwitchTFI when considering the fraction of differentially expressed drivers.

Despite these promising results, SwitchTFI comes with several limitations. First of all, SwitchTFI requires progenitor/offspring annotations as input. In cases where these cell state annotations do not come with the experimental procedure, they have to be inferred from the scRNA-seq data using trajectory inference tools, which may affect downstream results computed by SwitchTFI. Secondly, the quality of SwitchTFI’s results is strongly dependent on the quality of the input GRN. It is the starting point for the analysis during which edges are pruned from the GRN based on their predicted relevance to the cell state transition. Consequently, only regulatory subnetworks that are already present in the input GRN can be extracted as putative transition driver mechanisms.

For this article, we used the state-of-the-art tool Scenic to compute the baseline GRN, but inferring a high-quality GRN from scRNA-seq data is a complex problem that is not fully solved. For instance, one notable shortcoming of Scenic and other existing GRN inference methods is their stochasticity, meaning that several runs on the same data can yield different outputs [64]. To assess to which extent this stochasticity is inherited by SwitchTFI, we conducted a small robustness study where we ran SwitchTFI multiple times on different GRNs inferred by multiple Scenic runs on the same data (see “Methods” for details). Indeed, differences in GRNs inferred by Scenic lead to differences in transition GRNs computed by SwitchTFI, although we observe a small increase in robustness when comparing the edges of the different networks (see Additional file 1: Table S11).

Future versions of SwitchTFI would hence benefit from GRN inference algorithms with improved robustness to random bias. Possible pathways toward the design of such algorithms may be to adapt SwitchTFI’s FWER control mechanism or to use network enumeration techniques as previously suggested for robust disease module mining in protein-protein interaction networks [65]. Another interesting avenue for future work would be to develop a differential version of SwitchTFI to distinguish TFs and TF-target gene links that are relevant only to the specific transition from progenitors to offspring cells investigated by the user from those that, beyond this specific transition, are also relevant in other cell differentiation processes.

## Conclusions

While there is a plethora of computational methods to infer pseudotime annotations from scRNA-seq data, only few tools exist which can derive hypotheses on regulatory processes that drive transitions from progenitor to offspring cell states. With SwitchTFI, we address this gap by proposing an algorithm that takes TF-target links in a baseline GRN as elementary units of analysis and returns a filtered transition GRN containing only those edges whose associations with the cell state transition are statistically significant. A systematic benchmark on $\alpha$-cell, $\beta$-cell, and erythrocyte development datasets shows that, among all tested methods, SwitchTFI yields the best tradeoff between ptDE fraction and functional coherence of the predicted differentiation drivers; a case study in the context of T helper cell exhaustion shows that it can generate novel and biologically plausible hypotheses. SwitchTFI is interoperable with the scverse ecosystem [66], seamlessly interfaces with popular software for scRNA-seq data analysis such as Scanpy [67] and anndata [68], and is distributed as an easy-to-install Python package on Bioconda [69]: https://anaconda.org/bioconda/switchtfi.

## Methods

### SwitchTFI: input data

The input to SwitchTFI consists of three components:

- A scRNA-seq count matrix $X\in \mathbb {R}^{\mathcal {C}\times \mathcal {G}}$, where $\mathcal {C}$ and $\mathcal {G}$ are sets of cells and genes, respectively.
- A GRN $G=(\mathcal {G},\mathcal {E})$ that matches the biological context of the count matrix *X* or was inferred from it. *G*’s node set should match the set of genes $\mathcal {G}$ in the count matrix *X* and directed edges $(i,j)\in \mathcal {E}\subseteq \mathcal {G}\times \mathcal {G}$ connect TFs *i* to their target genes *j*.
- A partition $P \cup O = \mathcal{C}$ of the set of all cells $X$, quantified in *X*, into progenitor cells *P* and offspring cells *O*.

### SwitchTFI: data imputation with MAGIC

“Dropout” in scRNA-seq data refers to the problem that genes with non-zero biological expression are not detected due to limited sequencing sensitivity. Since dropout over-proportionally affects lowly expressed genes [70] and TFs are typically lowly expressed [71], it is particularly critical for SwitchTFI. Moreover, SwitchTFI’s edge-wise approach requires the co-expression of both TFs and their targets within the same cell. For each cell and each edge $e = (i, j)\in \mathcal {E}$ of the GRN *G*, this implies that

$$\begin{aligned} \textit{Prob}\left( \text {Edge}\ e\ \text {drops out}\right)= & \textit{Prob}\left( (\text {TF}\ i\ \text {drops out})\cup (\text {Target}\ j\ \text {drops out})\right) \\\ge & \max \left\{ \textit{Prob}\left( \text {TF}\ i\ \text {drops out}\right) ,\textit{Prob}\left( \text {Target}\ j\ \text {drops out}\right) \right\} \text {,} \end{aligned}$$

which further increases the relevance of the dropout problem for SwitchTFI.

To address this problem, we used the MAGIC imputation method [15] before weight fitting, which has been validated to be effective at mitigating dropout in scRNA-seq data to recover gene-gene relationships [15, 72]. MAGIC calculates an imputed expression matrix by performing data diffusion on a similarity-weighted *k*-nearest neighbor graph. A technical parameter *t* controls the depth of the diffusion process. Here, $t=1$ was used, which corresponds to the most conservative choice and has been shown to recover many true-positive gene-gene correlations while maintaining a low false positive rate [72] (note that, with $t=1$, zeros persist in the MAGIC-imputed data for cells *c* where a non-expressed gene *i* is not expressed in any of *c*’s neighbors in the *k*-nearest neighbor graph). Indeed, running SwitchTFI without MAGIC imputation leads to artifacts within the weights, where increased variance can be observed among edges where only few cells with TF-target co-expression are available for regression stump fitting (see Additional file 1: Fig. S8).

### SwitchTFI: weight fitting

Iterating over the edges $e = (i, j)\in \mathcal {E}$ of the GRN *G*, a weight $w_e \in [0.25,1]$ that indicates the relevance of the respective edge to the transition between the progenitor and offspring cell states is fit to each edge as follows: The expression vectors of the TF *i* and its target *j* are the column vectors $x_{\bullet ,i}\in \mathbb {R}^\mathcal {C}$ and $x_{\bullet ,j}\in \mathbb {R}^\mathcal {C}$ of the expression matrix *X*. A regression tree of depth 1 (regression stump) $T_e: \mathbb {R} \rightarrow \mathbb {R}$ is fit to predict the target expression $x_{\bullet ,j}$ from the TF expression $x_{\bullet ,i}$. The fitted regression stump is defined by a decision boundary $b_e \in [\min (x_{\bullet ,i}), \max (x_{\bullet ,i})]$, where $b_e$ is chosen such that it minimizes the mean squared error $|\mathcal {C}|^{-1} \sum _{c \in \mathcal {C}}^{c}(x_{c,j} - T_e(x_{c,i}))^2$. The prediction of the regression stump is defined as

1 $$\begin{aligned} T_e(x) =\left\{ \begin{array}{ll} \frac{1}{\left| L_e \right| } \sum _{c \in L_e} x_{c,j} & \text {if } x \le b_e \\ \frac{1}{\left| R_e \right| } \sum _{c \in R_e} x_{c,j} & \text {if } x> b_e \end{array}\right. \text {,} \end{aligned}$$

with $L_e = \{c \in \mathcal {C}\mid x_{c,i} \le b_e\}$ and $R_e = \{c \in \mathcal {C}\mid x_{c,i}> b_e\}=\mathcal {C}\setminus L_e$. The sets $L_e$ and $R_e$ correspond to a partition of the cells in the dataset, which is compared to the partition $\{P,O\}$ induced by the progenitor and offspring annotations. For this, the Jaccard index (defined as $JI(A, B) = \mid A \cap B \mid / \mid A \cup B \mid$ for sets *A* and *B*) is used to compute the edge relevance weight as follows:

2 $$\begin{aligned} w_e = \frac{\max \left\{ JI(P, L_e) + JI(O, R_e),\, JI(P, R_e) + JI(O, L_e)\right\} }{2} \end{aligned}$$

Note that the computation of $w_e$ corresponds to solving a trivial $2\times 2$ instance of a linear assignment problem, where the blocks of the two partitions are matched with respect to their Jaccard similarity. The edge weight $w_e$ is close to 1 if the partitions are similar and close to 0.25 if the partitions are dissimilar (see Proposition 1 below). Similarity of the inferred partition to the cell state partition (i.e., a high weight $w_e$) is interpreted as an indicator of high relevance of the edge *e* to the transition between cell states.

Direct regulation of transcription through TFs is mainly confined to the core of individual cells. Thus, when calculating $w_e$ for an edge $e = (i,j)$, we included only cells *c* with $x_{c,i},x_{c,j}>0$ in which both the TF *i* and its target *j* are expressed. For TF-target pairs that are both expressed only in a few cells, we observed the computed weights to behave capriciously (Additional file 1: Fig. S8). As explained above, this problem was mitigated by including data imputation with MAGIC [15] into SwitchTFI’s preprocessing workflow. Still, we recommend not including weights $w_e$ for edges $e = (i,j)$ in the analysis where *i* and *j* are co-expressed in fewer than 20% of all cells.

#### Proposition 1

(Range of $w_e$). The range of the edge relevance weights $w_e$ is [0.25, 1].

#### Proof

First, we assume without loss of generality that there is at least one progenitor and one offspring cell, meaning that $P\ne \emptyset \ne O$ (otherwise, the Jaccard indices in the definition of $w_e$ may be undefined). Since the maximum Jaccard index is 1, we clearly have $w_e\le 1$. If $P=L_e$ or $P=R_e$, it holds that $w_e=1$; and we have $w_e=0.25$ if $|P|=|O|$ and either $L_e=\mathcal {C}$ or $L_e=\emptyset$. It thus remains to be shown that $w_e\ge 0.25$ holds for all pairs of partitions $\{L_e,R_e\}$ and $\{P,O\}$ of $\mathcal {C}$ with $P\ne \emptyset \ne O$. With $a=|P\cap L_e|/|\mathcal {C}|$, $b=|P\cap R_e|/|\mathcal {C}|$, $c=|O\cap L_e|/|\mathcal {C}|$, and $d=|O\cap R_e|/|\mathcal {C}|$, we can rewrite Eq. 2 as $w_e=f(a,b,c,d)/2$, where

3 $$\begin{aligned} f(a,b,c,d)=\max \left\{ \frac{a}{1-d}+\frac{d}{1-a},\frac{b}{1-c}+\frac{c}{1-b}\right\} \text {,} \end{aligned}$$

$a+b+c+d=1$, and $0\le a,b,c,d<1$. Since $0\le d<1$, we know that $1-d\le 1$, which implies $1/(1-d)\ge 1$ and thus $a/(1-d)\ge a$. Analogous statements apply to the three other terms in the right-hand side of Eq. 3. This implies $f(a,b,c,d) \ge \max \{a+d,b+c\}$ and hence $f(a,b,c,d)\ge 0.5$, since otherwise, it would hold that $a+d<0.5$ and $b+c<0.5$, contradicting $a+b+c+d=1$. Hence, we have shown that $w_e=f(a,b,c,d)/2\ge 0.5/2=0.25$, as required.

### SwitchTFI: *P*-value calculation

Only the edges most relevant to the cell state transition should be retained in the GRN. For defining a sensible cut-off for the previously computed weights, we adopted a statistical view on them. For each edge *e*, its weight $w_e$ can be interpreted as the test statistic of a hypothesis test with the following null and alternative hypotheses:

- $H_0^{(e)}$: Edge *e* has no relevant explanatory power for the cell state transition from progenitor to offspring cells.
- $H_1^{(e)}$: Edge *e* has relevant explanatory power for the cell state transition from progenitor to offspring cells.

The distribution and dependency structure of the weights $w_e$ is unknown and we have to take into account the problem of multiple testing. Thus, we adopted the Westfall-Young permutation method [12] for calculating adjusted edgewise *P*-values. Again, we iterate over the edges $e = (i, j)\in \mathcal {E}$, where each edge is now associated with an already computed partition $\{L_e, R_e\}$ of the cells. Randomly permuting the progenitor/offspring labels of the cells that were used to calculate $w_e$ gives a new random cell partition $\{\tilde{P}, \tilde{O}\}$ and consequently a permutation weight

4 $$\begin{aligned} \tilde{w}_e = \frac{\max (JI(\tilde{P}, L_e) + JI(\tilde{O}, R_e),\, JI(\tilde{P}, R_e) + JI(\tilde{O}, L_e))}{2}. \end{aligned}$$

Repeating the permutation procedure $q\in \mathbb {N}$ times results in permutation weights $\tilde{w}_e^{(1)}, \ldots , \tilde{w}_e^{(q)}$ for all $e \in \mathcal {E}$, for which we define $w_{\max }^{(k)} = \max _{e \in \mathcal {E}} w_e^{(k)}$ for all $k \in \{1,\ldots , q\}$. The adjusted *P*-values are then computed as

5 $$\begin{aligned} P_e = \frac{1}{q}\sum \limits _{k=1}^q \left[ w_{\max }^{(k)} \ge w_e\right] \text {,} \end{aligned}$$

where $[\cdot ]$ is the Iverson bracket (i. e., $[\texttt{True}]=1$ and $[\texttt{False}]=0$). In general, if $P_l\le \alpha$ for all adjusted *P*-values, this method controls the

6 $$\begin{aligned} \text {FWER} = \textit{Prob}\left( \text {Reject at least one }H_0^{(l)} \mid \bigcap _{l = 0}^K H_0^{(l)}\right) \end{aligned}$$

at level $\alpha \in [0,1]$ for *K* simultaneously performed tests with null hypotheses $H_0^{(l)}$ and corresponding adjusted *P*-values $P_l$ [12]. Pruning all edges with $P_e> \alpha$ from *G* hence yields a cell state transition-specific GRN with controlled edge-level FWER, retaining only the most relevant edges for any kind of downstream analysis. Our recommendation is to use $\alpha = 0.05$, as this results in a concise and well-interpretable output.

### SwitchTFI: TF ranking

As an additional output, SwitchTFI returns a list of TFs, ranked by their relevance to the cell state transition. The ranking is computed by ordering the TFs according to their unweighted PageRank centrality [16] in the pruned GRN. Before the PageRank algorithm is run, the direction of the edges is reversed such that they are directed from targets to TFs. This way, the TFs most central in the topology of the GRN are found by the PageRank algorithm.

Alternatively, TFs can be ranked by their weighted outdegree in the transition GRN. For this, we compute edge-level scores

7 $$\begin{aligned} s_e = -\log _{10}(P_e)\cdot w_e \end{aligned}$$

that combine the JI-based edge weights $w_e$ and the Westfall-Young *P*-values $P_e$ and then obtain the weighted outdegree of a TF *i* by summing up the scores $s_e$ of its outgoing edges $e=(i,j)$ in the transition GRN.

### Datasets

The murine pancreatic endocrinogenesis dataset is part of the scRNA-seq study presented by Bastidas-Ponce et al. [19, 20]. It contains the transcriptomic profiles of pancreatic epithelium cells sampled at embryonic day 15.5. Cell-level annotations of developmental cell states are provided. For analysis with SwitchTFI, the dataset was subset into two separate datasets, each containing only cells from the progenitor cell state (pre-endocrine) and one offspring cell state ($\alpha$-cell or $\beta$-cell).

The hematopoiesis scRNA-seq dataset was created and analyzed by Paul et al. [21, 22]. It contains the transcriptomic profile of myeloid progenitors differentiating towards seven distinct differentiation fates. Subsetting to the erythrocyte trajectory and merging the fine-grained cell state annotations that come with the dataset yielded the progenitor/offspring structure needed for SwitchTFI analysis.

For scalability tests, we used a large-scale dataset (85010 cells) from a cell reprogramming experiment [50, 51], where mouse embryonic fibroblasts (MEFs) were reprogrammed to induced endoderm progenitors (iEPs). We defined progenitor and offspring populations based on the reprogramming day annotations and an MEF/iEP identity score analysis provided by the authors (day 0 and 3: progenitors; day 6 onwards: offspring; see Figure 1f in [50] for justification of the cutoff). However, we largely disregarded the biological context of this dataset since it was used exclusively for scalability tests.

For our T helper cell exhaustion case study, we used a Th1 dataset from acute and chronic LCMV infection models [52]. We used the Th1 subset annotations and diffusion maps provided with the dataset. For running SwitchTFI, early and terminally differentiated Th1 cells from the acute infection model were used as progenitor and offspring cells, respectively.

### Preprocessing of scRNA-seq data

The best practices according to Heumos et al. [73] were used as a guideline. They include filtering with respect to quality on the cell and gene level, correcting for ambient RNA with the SoupX method [74], and data normalization. The quality control steps were performed independently of the downstream method. All subsequent processing steps, such as data normalization or highly variable gene selection, were performed following the tutorials provided with each method. The individual steps are detailed and motivated in Additional file 1: Section 2.

### GRN inference

For each dataset, a GRN was inferred with the Scenic workflow presented in [13]: First, a basic GRN is inferred with GRNBoost2 [75], which is then pruned based on *cis*-regulatory motif analysis. GRNBoost2 employs gradient boosting for more efficient GRN inference compared to the seminal GENIE3 method [76], on which it is based. Still, the resulting GRN is coexpression-based and can contain many false positives and indirect targets. By pruning targets that lack motif support, the remaining edges constitute a biologically informed GRN. Owing to the inherent stochasticity of GRNBoost2, the output of Scenic varies greatly between individual runs. To mitigate this problem, Scenic was run $n=18$ times and a combined GRN was constructed from the edges that appeared in $k \ge 9$ GRNs. To perform the computations, the Python implementation pySCENIC (https://github.com/aertslab/pySCENIC) of the Scenic method was used.

### Validation strategy

For validation steps solely concerning SwitchTFI, the input data of SwitchTFI was used to compute the validation metrics. For the method comparison, the scRNA-seq data was preprocessed according to the specifications of each method. This assures a fair basis for comparison, since the competitor methods assume non-imputed input data. The ptDE *q*-values were computed on non-imputed, total count normalized, and $\log$-transformed data, as required by switchde [24].

The competitor methods were run as described in the tutorials that come with the respective Python packages. Also the preprocessing was adopted accordingly. The software versions and tutorials that were used are listed in Additional file 1: Table S1. Small adjustments have been made to fit our test setting. As mentioned in “Background”, for analyses with spliceJAC, the number of considered genes is limited by the minimal number of cells within a cell state. In their tutorial, only 50 genes were included. Here, 300 and 400 genes were included for the analyses of the pre-endocrine-$\alpha$ and -$\beta$ transition. DrivAER relies on a predefined family of gene sets that is usually derived from a third-party bioinformatics database. Here, the sets of targets of individual TFs in the GRN inferred with Scenic were used to define such a family, enabling a fair comparison against SwitchTFI.

### Pseudotime inference

The pseudotime value assigned to each cell was computed using the algorithm presented as a part of the Palantir method [5]. It is based on shortest path distances from a user-defined early cell in a graph embedding of the scRNA-seq data. The root cell was chosen according to the expression of known marker genes. For the pancreatic endocrinogenesis dataset, the cell with the highest Fev expression among the pre-endocrine cells was used; for the hematopoiesis dataset, we used the progenitor cell with the lowest Gata1 expression. The pseudotime was computed on total count normalized, and log-transformed data.

### Gene expression trend computation

In accordance with Palantir [5] and CellRank [6], a generalized additive model (GAM) [77] with preprocessed and Magic-imputed data as an input was used to compute gene expression trends over pseudotime. In general, GAMs model the relationship between a univariate response variable, here the gene expression, and (multiple) predictor variables, here the pseudotime. Using the LinearGAM class of the pyGAM package [78], a GAM with a Normal error distribution and an identity link was fit. For the functional form, a spline term consisting of four basis functions of degree two was used. The prediction of the GAM for evenly spaced pseudotime values was used for visualizing gene expression trends. Moreover, 0.95-confidence intervals for the predictions as computed by pyGAM are shown around the trend.

### Differential expression testing

Testing for differential expression of genes along a pseudotime trajectory was performed using switchde [24]. In switchde, a switch-like expression pattern of a gene is quantified by fitting a sigmoid function to the gene expression in pseudotime. The parameters of the sigmoid are determined as maximum likelihood estimates, where the actual optimization task is performed with L-BFGS-B [79], a standard nonlinear optimization algorithm. A likelihood ratio test between the sigmoid model and a constant-expression model yields *P*-values for differential expression. Furthermore, switchde provides an extension to fit sparse scRNA-seq data, which we used for non-imputed data. The Benjamini-Hochberg procedure was used to compute *q*-values that are corrected for multiple testing. To analyze differential expression of TFs in progenitor Th1 cells between the acute and the chronic LCMV infection models and between progenitor and offspring cells in the acute infection model, we used the ZINB-WaVE [56] extension of edgeR [57] which accounts for zero inflation in the scRNA-seq data for the weakly expressed TFs. Subsequently, the obtained *P*-values were adjusted for multiple testing using Benjamini-Hochberg correction.

### Transition GRN validation

The transition GRN $G^T=(\mathcal {G}^T,\mathcal {E}^T)\subseteq G$ computed by SwitchTFI is a subnetwork of the input GRN $G= (\mathcal {G},\mathcal {E})$, where $\mathcal {E}^T=\{e\in \mathcal {E}\mid P_e\le 0.05\}$ and $\mathcal {G}^T=\{i\in \mathcal {G}\mid \exists e\in \mathcal {E}^T: i\in e\}$ (that is, $G^T$ is the subnetwork of *G* induced by the edges $e\in \mathcal {E}^T$ with significant adjusted *P*-values). To assess connectivity of $G^T$, we randomly generated subnetworks of *G* by sampling $|\mathcal {E}^T|$ edges $e \in \mathcal {E}$ uniformly at random without replacement and then taking the edge-induced subgraph. Repeating this procedure $q\in \mathbb {N}$ times yielded subnetworks $(\mathcal {G}^{(k)}, \mathcal {E}^{(k)}) = G^{(k)}\subseteq G$ for $k=1,\ldots ,q$. For each of those subnetworks $G^{(k)}$, let $\mathcal {C}^{(k)} = \{C_{1}^{(k)}, \ldots , C_{c_{(k)}}^{(k)}\}$ with $C_{i}^{(k)}\subseteq \mathcal {G}^{(k)}$ be the set of disjoint node sets that induce the $c_{(k)}$ weakly connected components of $G^{(k)}$. The connectivity score of $G^{(k)}$ is then computed as follows:

8 $$\begin{aligned} CS^{(k)} = \sum \limits _{r=1}^{c_{(k)}} \left( \frac{|C_r^{(k)}|}{|\mathcal {G}^{(k)}|}\right) ^2 \end{aligned}$$

The score $CS^T$ for the transition GRN is computed similarly. The corresponding empirical *P*-value is:

9 $$\begin{aligned} P_{CS^T} = (q+1)^{-1}\cdot \left( 1+\sum \limits _{k=1}^q \left[ CS^{(k)} \ge CS^T\right] \right) \end{aligned}$$

### Gene set enrichment analysis

Gene set enrichment analysis for the top 10 predicted driver genes was performed using the Enrichr webtool [25, 26]. We ranked enriched terms according to their adjusted *P*-value, which is computed by Enrichr with Fisher’s exact test. The enrichment was tested on the gene set libraries Gene Ontology Biological Process and Cellular Component [80] and Reactome [81].

### Functional coherence testing

Analyses of the functional and genetic coherence of the top 20 putative driver genes was performed with DIGEST [49]. DIGEST takes an unordered set of genes as an input. For each gene in the set, the set of its functional annotations is retrieved from a reference database. Available databases are the Gene Ontology [80] (Biological Process, Cellular Component, and Molecular Function) and the Kyoto Encyclopedia of Genes and Genomes [82]. The average Jaccard index for all pairs of gene-wise annotation sets is computed as the functional relevance score. To compute empirical *P*-values for the scores, randomized gene sets are created according to a random background model. Here, the fully randomized mode (genes are drawn uniformly at random without replacement) implemented by DIGEST was used.

### Robustness study

Given an annotated scRNA-seq count matrix *X*, we computed 18 GRNs $G_r = (\mathcal {V}_r, \mathcal {E}_r)$, $r = 1, \ldots , 18$, by running Scenic 18 times on *X*. Then we ran SwitchTFI with PageRank- and outdegree-based TF ranking on these GRNs, leading to 18 transition GRNs $G_r^\star = (\mathcal {V}_r^\star , \mathcal {E}_r^\star )$ and 18 sets $T_r$ of top 10 putative driver TFs in $G_r^\star$. In the following, $T_r$ is interpreted as an unordered set. For each type of set $S_r\in \{\mathcal {V}_r, \mathcal {E}_r,\mathcal {V}_r^\star , \mathcal {E}_r^\star ,T_r\}$, similarities $s(S_1, \ldots , S_{18})$ are computed as the average pairwise Jaccard index

10 $$\begin{aligned} s(S_1, \ldots , S_{18}) =\left( {\begin{array}{c}18\\ 2\end{array}}\right) ^{-1}\sum \limits _{r=1}^{17}\sum \limits _{r^\prime =r+1}^{18} JI(V_r, V_{r^\prime }). \end{aligned}$$

Comparing the similarity values obtained for the input GRNs $G_r$ and for the transition GRNs $G_r^\star$ allows to assess to which extent Scenic’s lack of robustness with respect to random bias is inherited by SwitchTFI.

### Scalability analysis

Scalability of the compared methods was assessed by running each method on subsets of different size of the cell reprogramming dataset [50, 51]. The count matrix was subset to the 10000 most variable genes. The baseline GRNs required as input by DrivAER and SwitchTFI were inferred with Scenic on the same number of cells as used in the respective benchmark run. Since the runtime of DrivAER and SwitchTFI not only depends on the number of cells *n* but also on the number of edges *m* in the baseline GRN, we defined two benchmark grids as follows:The relative GRN sizes $m(n)=\text {``small''}$, $m(n)=\text {``medium''}$, and $m(n)=\text {``large''}$ correspond to 25%, 50%, and 75% of the total number of edges in the baseline GRN for *n* cells. This definition is motivated by the observation that, across GRN inference runs with Scenic, the number of edges that occur in at least 50% of the resulting GRNs is typically around 50% of the number of edges in the individual GRNs. Benchmark runs were carried out on a compute node hosted at the Erlangen National High Performance Computing Center (NHR@FAU), equipped with two Intel Xeon Gold 6326 CPUs (“Icelake”, 2 x 16 cores, 2.90 GHz base frequency). We allocated one CPU per job, except for GRN inference, where all 32 cores were used by Scenic’s parallelized implementation.

## Supplementary Information

Additional file 1. Contains an in-depth review of competitor methods, details on scRNA-seq data preprocessing, supplementary information on hyperparameter selection, and supplementary results

## Data Availability

The source code of the SwitchTFI method is available at https://github.com/bionetslab/SwitchTFI and on Zenodo [83] under the terms of the GNU General Public License v3.0. An easy-to-install packaged version of SwitchTFI is available on Bioconda at https://anaconda.org/bioconda/switchtfi. Source code to reproduce the results reported in this article is available at https://github.com/bionetslab/SwitchTFI-validation and on Zenodo [84] under the terms of the GNU General Public License v3.0. The murine pancreatic endocrinogenesis scRNA-seq dataset originally published in [19, 20] was obtained through the scvelo.datasets.pancreas() function of the scVelo Python package [6]. The hematopoiesis scRNA-seq dataset originally published in [19, 20] was obtained through the scanpy.datasets.paul15() function of the Scanpy Python package [67]. The cell reprogramming scRNA-seq dataset originally published in [50, 51] was obtained through the cellrank.datasets.reprogramming_morris() function of the CellRank Python package [6]. The T helper cell scRNA-seq dataset used for our case study was obtained from NCBI Gene Expression Omnibus [52].
