# Supplementary material for: SwitchTFI: identifying transcription factors driving cell differentiation
Source: Genome Biol. 2025 Dec 2;26:410. doi: 10.1186/s13059-025-03876-0 (PMC12673684; doi:10.1186/s13059-025-03876-0)
Supplement: Supplementary file 1 — Additional file 1. Contains an in-depth review of competitor methods, details on scRNA-seq data preprocessing, supplementary information on hyperparameter selection, and supplementary results [file 13059_2025_3876_MOESM1_ESM.pdf]

SwitchTFI: identifying transcription factors driving cell differentiation

Paul Martini, Anne Hartebrodt, Gustavo P. de Almeida, Carl-Philipp Hackstein, Dietmar Zehn, David B. Blumenthal

Supplementary information

Contents

1 In-depth review of competitor methods 1

2 Details on scRNA-seq data preprocessing 3

3 Supplementary information on hyperparameter selection 3

4 Supplementary results 5

1 In-depth review of competitor methods

Palantir. Palantir [Setty et al., 2019] models cell differentiation as a Markov process on a nearest-neighbor graph embedding of the scRNA-seq data. Starting from a user-defined initial cell a shortest-path based pseudotime is computed, which is used to determine the directionality of the edges in the graph embedding. Trajectories are identified by associating them with terminal states of the Markov chain. For each cell, the probability that a Markov process starting from that cell ends up in a specific terminal state is called its branch probability. The probability distribution over the trajectorial branches then serves as a soft assignment of each cell to the identified branches. For identifying key genes that drive differentiation along a certain branch towards its associated terminal state, Palantir correlates branch probabilities and gene expression using the Pearson correlation coefficient.

CellRank. CellRank [Lange et al., 2022] improves upon Palantir by integrating RNA velocity [La Manno et al., 2018, Bergen et al., 2020] information for inferring the directionality of the edges in the graph embedding, rendering the need for a user-defined initial cell superfluous. Again, driver genes are computed using the Pearson correlation between branch probabilities and gene expression. Both Palantir and CellRank have been proven to work well for gaining a functional understanding of cell differentiation from scRNA-seq data, but do not incorporate mechanistic information to identify genes which drive the cell differentiation.

DrivAER. Given a scRNA-seq count matrix, a family of gene sets, and a phenotype vector of interest DrivAER [Simon et al., 2020] combines a deep count autoencoder network (DCA) as proposed by Eraslan et al. [2019] for denoising and imputation of scRNA-seq data with a random forest (RF) model to compute relevance scores for the individual gene sets. The DCA is trained on each of the gene sets separately, such that the output of the bottleneck layer yields an embedding of the cells in low-dimensional latent space for each gene set. With these low-dimensional representations as the input and the phenotype vector as the target, a RF classification or regression model is deployed, depending on the scale of the target vector entries. The out-of-bag accuracy

score serves as the relevance score for each gene set. By defining annotations of consecutive cell stages during differentiation or a pseudotemporal ordering as the phenotype of interest, the relevance score can reveal gene sets that are key to the underlying cellular process. TFs that drive differentiation can be found by defining the family of gene sets as a collection of sets of target genes of known TFs. DrivAER works well for identifying relevant TFs but cannot recover which individual TF-target gene links are relevant. This is because the embedding on whose basis the relevance scores are computed has a lower dimension (2D per default) than the number of target genes it is computed from. Individual TF-target gene relationships are lost.

SpliceJAC. SpliceJAC [Bocci et al., 2022] assumes a scRNA-seq count matrix with spliced and unspliced counts and a cell annotation vector containing the respective cell states as an input to infer cell state-specific gene-gene regulatory interactions and putative driver genes, using a dynamical system model. The interconnection between genes and mRNA splicing dynamics is modeled with a system of nonlinear first-order ordinary differential equations. Non-linearity appears in the gene regulation function, which represents the regulatory effects between genes with the spliced counts as the function argument. By assuming the distinct cell states to be attractors in the phase space of the underlying dynamical system and by assuming the gene regulation function to be linear when close enough to an attractor, the gene regulation function is considered to be linear for each distinct cell state. Further, by assuming the steady states of the dynamical system are fixed points of the corresponding system of differential equations, finding a solution reduces to solving a linear regression problem for each cell state. For the solution to be unique, the number of considered genes must be limited by the number of cells within a given cell state. This poses a severe limitation, since an extensive preselection of the considered genes is required. In the tutorial published with the spliceJAC Python package, only 50 out of 27998 possible genes are used for GRN inference and transition driver gene identification. Genes that are critical to the transition between cell states are found by analyzing the eigenspace of the Jacobian matrix associated with the differential equations of the starting cell state. The path displacement vector, which connects the starting and final cell state in gene space, is decomposed into a linear combination of the eigenvectors with the top k largest corresponding eigenvalues. Those are regarded as unstable transition directions, such that the sum of a genes loadings in the eigenvector projection onto the transition path can be interpreted as its instability score. Genes with large instability score are thus identified as transition driver genes. Since spliceJAC focuses on individual genes, TF-target gene relations that drive differentiation cannot be identified. To mitigate this limitation, spliceJAC also provides a GRN comprised of marker genes for the initial cell state and the top transition driver genes as the vertices and the predicted interactions between them as edges. Yet, it is unclear how sensible cutoffs for the vertices and edges to be included in the GRN should be defined. Further, the edges in the GRN are inferred without utilizing transition-specific information and are thus not necessarily relevant to the cell state transition.

Versions, access dates, and tutorials used for this study. The versions and access dates of the competitor methods' used for this study are listed in Table S1, along with links to the respective tutorials.

Table S1. Competitor methods, all accessed on June 14, 2024.

	Version	GitHub
CellRank	2.0.4	https://github.com/theislab/cellrank
spliceJAC	0.0.1	https://github.com/federicobocci/spliceJAC
DrivAER	0.0.2	https://github.com/lkmklsmn/DrivAER

	Tutorial
CellRank	https://cellrank.readthedocs.io/en/latest/notebooks/tutorials/estimators/700_fate_probabilities.html
spliceJAC	https://splicejac.readthedocs.io/en/latest/notebooks/Transitions.html
DrivAER	https://colab.research.google.com/drive/1zrQ7l3Orz7h-eGEX7MHRIBTTXzL_vu9O#scrollTo=VzAzfdHZrOWz

2 Details on scRNA-seq data preprocessing

Quality control. The best practices for scRNA-seq data preprocessing according to Heumos et al. [2023] were adopted as follows: Firstly, low-quality cells were filtered. The criteria for filtering are: (1) A low number of detected genes per cell. (2) A low number of counts per cell. (3) A disproportionally high percentage of the total counts concentrated on a few, say ≤ 20, genes. (4) A high fraction of counts from mitochondrial genes. A cell was filtered if it deviates by more than 5 median absolute deviations (mad) from the median of all cells with respect to criteria (1), (2) and (3) or more than 3 mads with respect to (4). Jointly considering criteria (1-4) reduces the risk of confounding a genuine cellular signal with low quality. The mean absolute deviation of a data vector $x \in \mathbb{R}^n$ is defined as $\text{mad} = \text{median}_{i=1,\ldots,n}(x_i - \text{median}(x))$. Also, cells with more than 8% mitochondrial counts were filtered. Secondly, correction for ambient RNA was performed with the SoupX method [Young and Behjati, 2020]. Lastly, quality control was also performed in the gene dimension. Genes that do not appear in at least 10 cells are considered uninformative and are filtered. Resulting dataset sizes are provided in Table S2.

Table S2. Dataset sizes (number of cells, number of genes).

Dataset	before quality control	after quality control
Pre-endocrine-α	1073, 27998	904, 10944
Pre-endocrine-β	1183, 27998	1002, 11123
Erythrocytes	1262, 3451	1130, 3010

Further method-specific preprocessing. After quality control, the count matrix was further processed according to method-specific workflows. We carried out the following steps, following the tutorials provided with each method as guidelines for the competitor methods (see Table S1):

- SwitchTFI: We normalize each cell by total counts over all genes and log-transformed the normalized count matrix ($x = \log(x+1)$). Moreover, MAGIC imputation was applied as an integral step of the SwitchTFI workflow.
- CellRank: We applied scVelo's [Bergen et al., 2020] `scvelo.pp.filter_and_normalize()` function. It removes genes with fewer than 20 spliced and unspliced counts, normalizes each cell by total counts over all genes, identifies and marks the top 2000 highly variable genes (without subsetting the data to these genes), and applies a log-transformation to the expression matrix ($x = \log(x+1)$).
- SpliceJAC: We applied the same preprocessing steps as for CellRank, except that the dataset was subset to include only the top 2000 highly variable genes.
- DrivAER: This method was provided with the quality-controlled count matrix directly, as it handles all downstream processing internally.

3 Supplementary information on hyperparameter selection

Tables S3, S4, and S5 show the sizes of the transition GRN for different choices of the FWER threshold $\alpha \in \{0.05, 0.1, 0.2, 0.5\}$. Stricter thresholds yield smaller, more concise transition GRNs, whereas more lenient thresholds permit the inclusion of more, possibly less relevant edges. Further we examined the similarity among the sets of top $k = \{1, 5, 10, 15, 20\}$ putative driver genes across $\alpha \in \{0.05, 0.1, 0.2, 0.5\}$. The average pairwise Jaccard indices of the sets are listed in Table S6 (averaged over α with k fixed). It can be seen that the driver TF ranking is not greatly affected by the choice of α. This means that the same TFs are central in the topology of the transition GRN, regardless of its size. The rankings computed with PageRank centrality are more sensitive to changes of α, while the arguably simpler outdegree centrality can be observed to yield

slightly more robust results. The choice of centrality measure is ultimately dictated by the aim of the downstream analyses. The notion of centrality and its underlying assumptions should fit the subject of study. Thus, other centrality measures such as eigenvector, katz, closeness, betweenness and vote rank as well as the option to set edge weights and change the directionality of edges are available with SwitchTFI's Python implementation.

Table S3. Sizes of transition GRN for α-cell transition data for different FWER thresholds.

FWER threshold	# vertices	# TFs	# targets	# edges
1.0 (no pruning)	2104	151	2003	2732
0.50	348	42	316	434
0.20	325	39	296	402
0.10	303	31	281	382
0.05	284	28	265	359

Table S4. Sizes of transition GRN for β-cell transition data for different FWER thresholds.

FWER threshold	# vertices	# TFs	# targets	# edges
1.0 (no pruning)	2267	150	2175	3016
0.50	596	31	575	710
0.20	548	28	529	650
0.10	531	28	512	626
0.05	531	28	512	626

Table S5. Sizes of transition GRN for erythrocyte differentiation data for different FWER thresholds.

FWER threshold	# vertices	# TFs	# targets	# edges
1.0 (no pruning)	1008	60	984	1961
0.50	410	35	389	550
0.20	381	30	363	506
0.10	337	27	322	443
0.05	335	27	320	436

Table S6. Average pairwise Jaccard indices of top-k TFs across FWER thresholds (rounded to 4 decimals).

dataset	centrality measure	$k = 1$	$k = 5$	$k = 10$	$k = 15$	$k = 20$
α-cell transition	PageRank	1.0	0.8334	0.8485	1.0	0.8194
	outdegree	1.0	1.0	0.9091	1.0	1.0
β-cell transition	PageRank	1.0	1.0	0.9091	0.8591	0.8485
	outdegree	1.0	1.0	1.0	1.0	0.9365
erythrocyte differentiation	PageRank	1.0	0.8334	0.8485	1.0	0.9091
	outdegree	1.0	1.0	0.8788	1.0	0.9365

4 Supplementary results

Fig. S1 presents scatter plots of TF versus target gene expression, each overlaid with the corresponding regression stump fitted during SwitchTFI's weight fitting step. For each dataset, we show the five edges with the highest and lowest weights. Again, highly weighted edges display clear cluster structures in the cell state annotations, which align well with the partition induced by the regression stump's decision boundary. This structure is not observed for the edges with low weights.

Fig. S2 shows the results obtained when using SwitchTFI to analyze the pre-endocrine to α-cell transition in the pancreatic endocrinogenesis data [Bastidas-Ponce et al., 2019]. The results confirm SwitchTFI's good performance observed for the pre-endocrine β-cell transition reported in the main article. Again, the fraction of ptDE edges and vertices is significantly increased in the transition GRN over the Scenic GRN (Fig. S2A, B). Also, the joint score for the number and size of connected components can again be observed to be significantly higher for the transition GRN compared to a random background model (Fig. S2C). Gene set enrichment for the top 10 putative driver genes revealed terms related to regulation of transcription and endocrinogenesis (Fig. S2D). Moreover, SwitchTFI compares favourably to the competitor methods also on the α-data (Fig. S2E-G).

Fig. S3 shows additional results for the erythrocyte differentiation dataset [Paul et al., 2015]. Here, the top 10 driver genes were ranked according to their weighted outdegree in SwitchTFI's transition GRN. Figure 6 in the main document suggests that this yields slightly more relevant results for the erythrocyte differentiation data. For the top 10 driver genes predicted by SwitchTFI, their rapidly increasing or decreasing expression trends over pseudotime are displayed in Fig. S3A. For the set of top 10 driver genes (Fig. S3B), the most significantly enriched terms relate directly to hematopoiesis or more generally to the regulation of transcription (Fig. S3C). For the top 10 putative driver genes, biologically validated evidence for their relevance to erythrocyte development can be found. E.g., it is well known that Gata1 and Gata2 are involved in gene regulation during erythropoiesis [Ohneda and Yamamoto, 2002]. While Gata2 is highly expressed in hematopoietic progenitors, erythroid differentiation is characterized by a switch to increased Gata1 expression [Suzuki et al., 2013]. This switch from Gata2 to Gata1 is clearly visible in the gene expression trends in Fig. S3A.

The top 20 putative driver genes from each of the compared methods used for the method comparisons reported in Fig. S2 and in Figure 5 in the main document are listed in Table S7. They are ordered by decreasing predicted relevance as differentiation driver genes. The detailed results of the functional coherence analysis with DIGEST are listed in Tables S8 to S10. Lastly, the detailed results of the robustness study mentioned in the Discussion of the main document are given in Table S11.

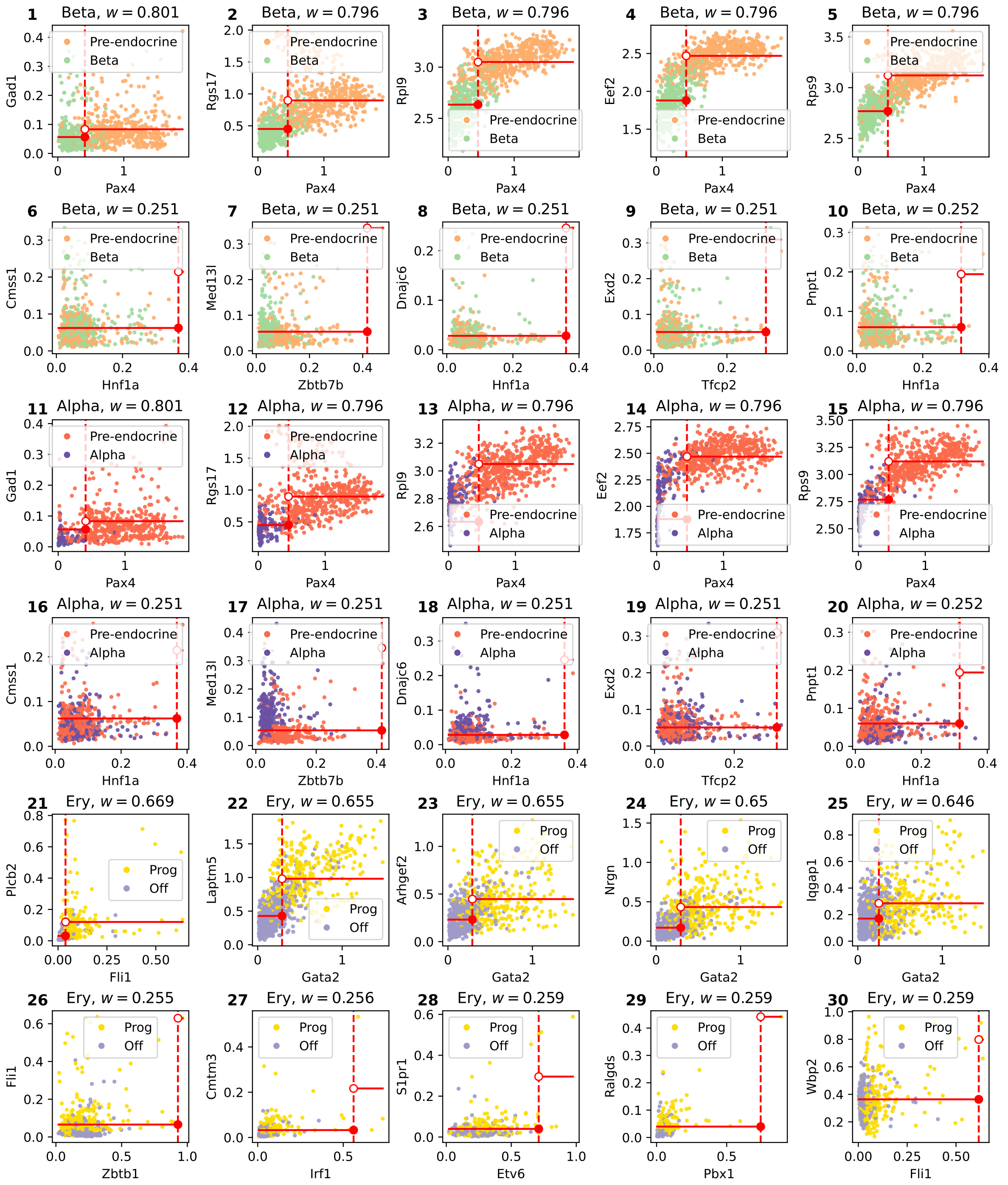

Fig. S1. Gene expression (with MAGIC imputation) of TFs (x-axis) and target genes (y-axis) for the cells in the scRNA-seq datasets. Panels (1–5), (11–15), and (21–25) correspond to the five highest weighted edges for the β-cell, α-cell, and erythrocyte development datasets, respectively. The corresponding lowest-weighted five edges are shown in panels (6–10), (16–20), and (26–30). The regression stump fit during SwitchTFI's weight fitting step is shown in red.

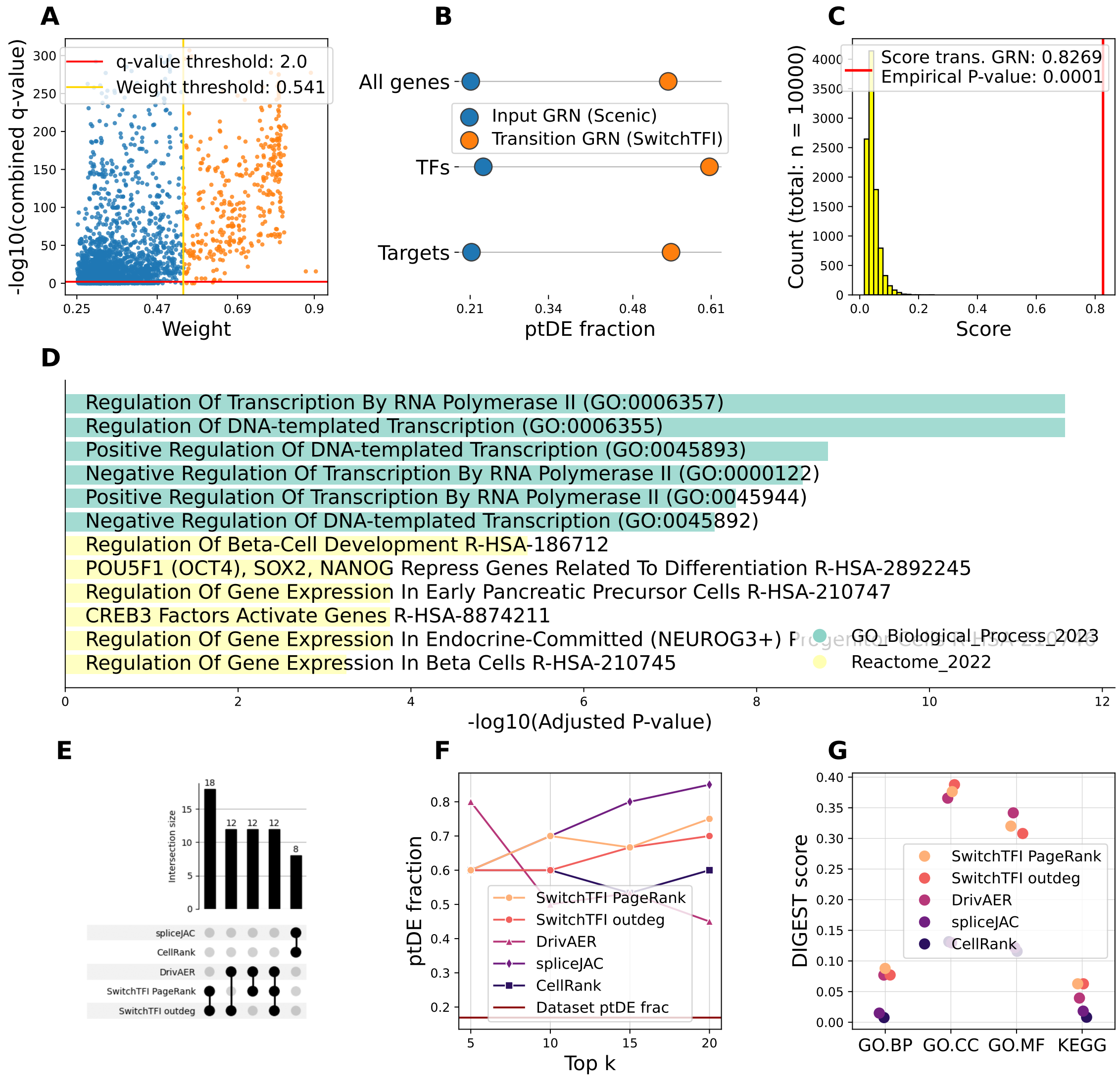

Fig. S2. Additional results for the pre-endocrine α-cell transition data. (A) Edge weights vs. combined ptDE q-values of the unpruned Scenic GRN. Combined q-values are $-\log_{10}(\cdot)$-transformed. (B) Fraction of ptDE genes, TFs and targets in the Scenic and SwitchTFI GRN. (C) Histogram of the connected component scores of randomly sampled subnetworks of the Scenic GRN. The transition GRN's score is visualized as a red line. (D) Gene set enrichment results for the top 10 putative α driver TFs. (E–G) Method comparison results, compare Figure 5 in the main document.

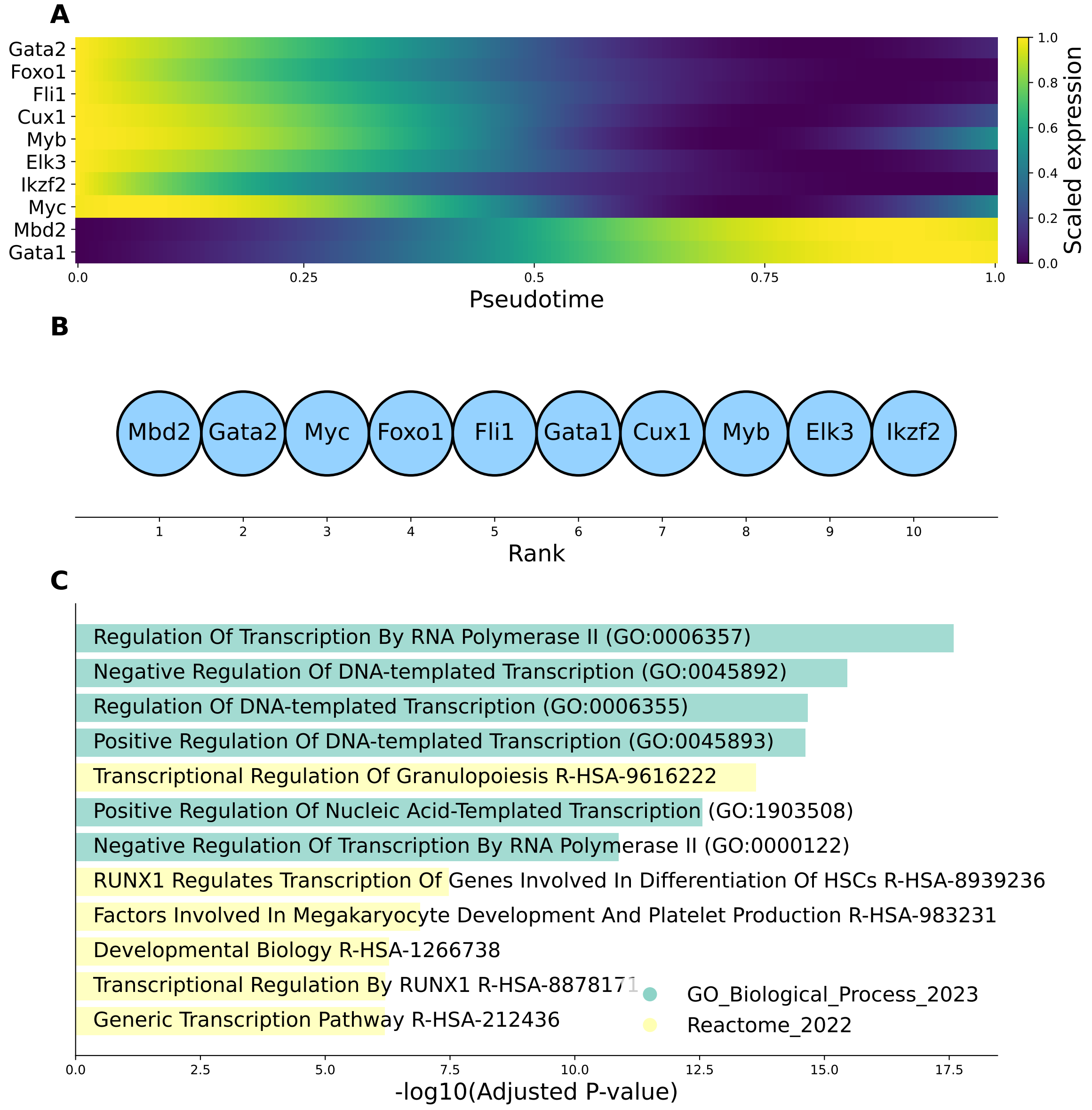

Fig. S3. Additional results for the erythrocyte differentiation dataset. (A) Expression trends in pseudotime of the top-10 TFs. (B) Top 10 putative driver TFs ranked by score weighted outdegree in the transition GRN. (C) Gene set enrichment results for the top 10 putative erythrocyte differentiation driver TFs.

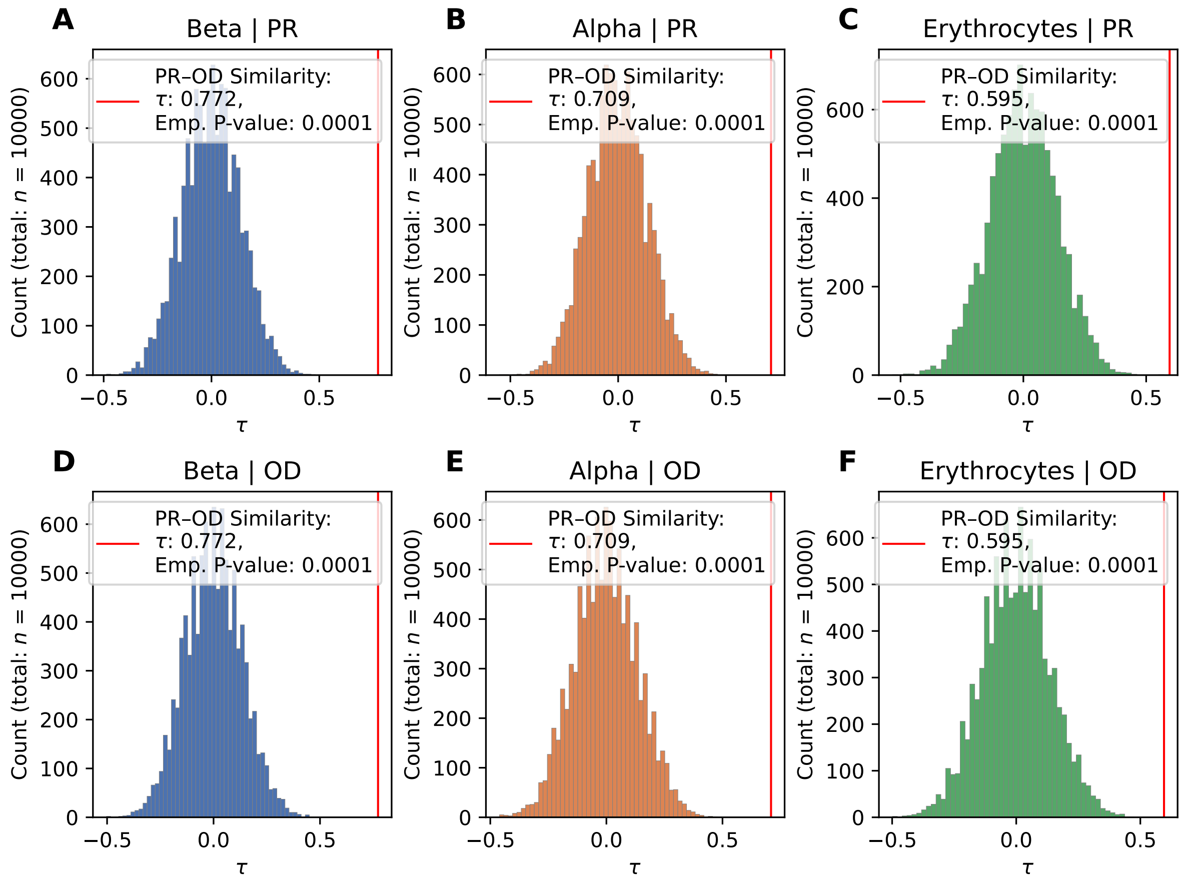

Fig. S4. Comparison of TF rankings obtained with PageRank (PR) and outdegree (OD) centrality to random permutations of all TFs in the transition GRNs (α- and β-cell development dataset: 27 TFs; erythrocyte development dataset: 28 TFs). Each panel shows the null distribution of Kendall's τ rank correlation coefficients obtained by comparing the PR (first row) or OD (second row) rankings with 10,000 random TF rankings for each dataset. The red line indicates Kendall's τ between PR and OD rankings. In all cases, the observed correlation is significantly higher than expected by chance, confirming that the similarities between PR and OD rankings are no artifacts of the small TF numbers in the transition GRNs.

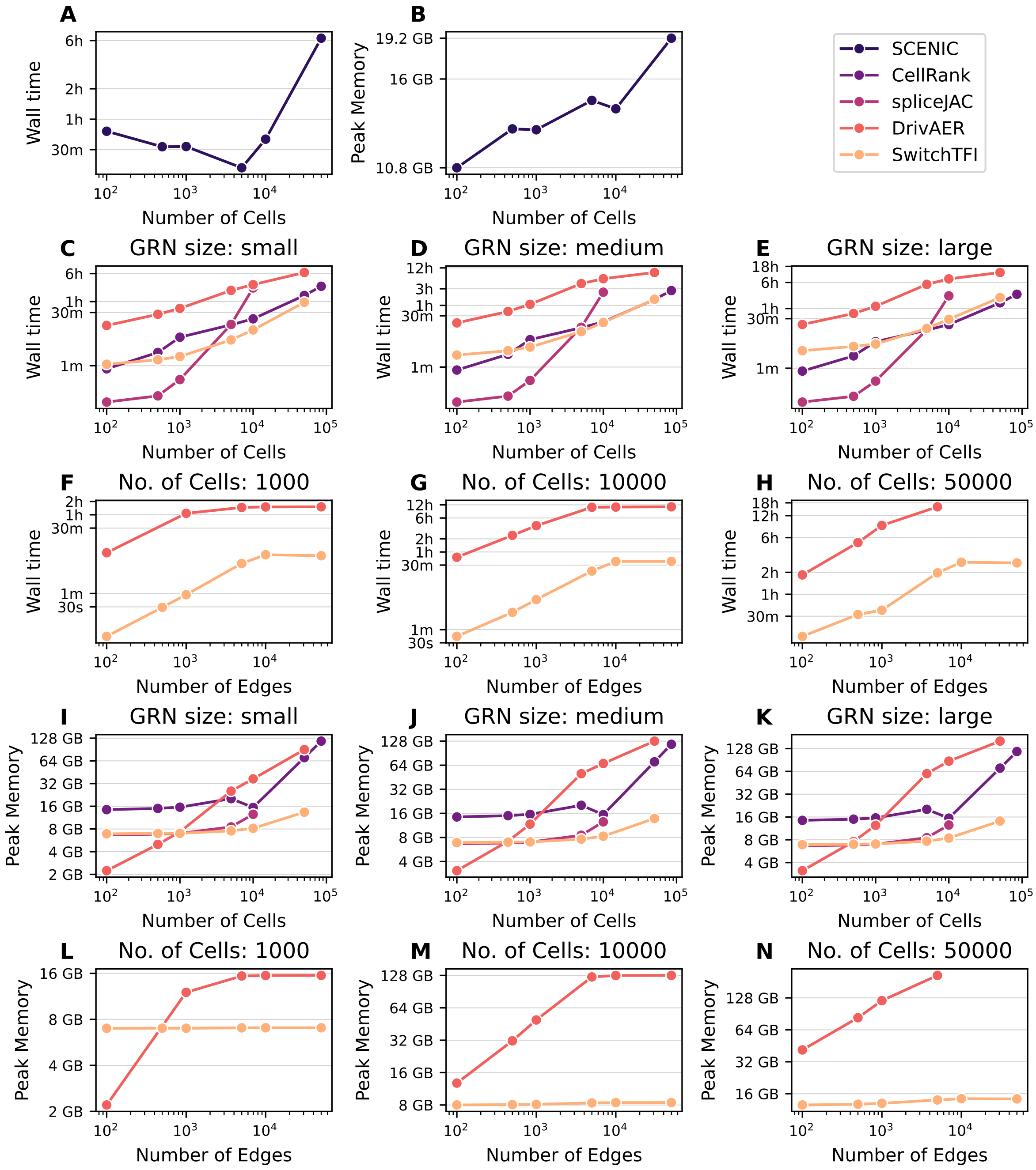

Fig. S5. Wall time and memory requirements on subsets of the reprogramming dataset by Biddy et al. [2018]. (A, B) Results for inference of a baseline GRN with SCENIC. The initially decreasing wall time in (A) results from issues in the parallelization of SCENIC's GRNBoost2 [Moerman et al., 2019] subroutine, which does not always use all CPUs. (C–H) Wall times for fixed GRN sizes and varying numbers of cells (C–E) and fixed numbers of cells and varying GRN sizes (F–H). (I–N) Memory requirements for fixed GRN sizes and varying numbers of cells (I–K) and fixed numbers of cells and varying GRN sizes (L–N). Due to a 24-hour time limit on our HPC system, GRN inference on the full dataset (85,010 cells) could not be completed. Consequently, DrivAER and SwitchTFI results are not available for this setting. Missing results for spliceJAC are also due to runtimes exceeding 24 hours. DrivAER additionally failed with 50,000 cells and GRNs of 10,000 and 50,000 edges because of out-of-memory errors. Results for spliceJAC and CellRank are identical in C–E and I–K because these methods do not use a GRN.

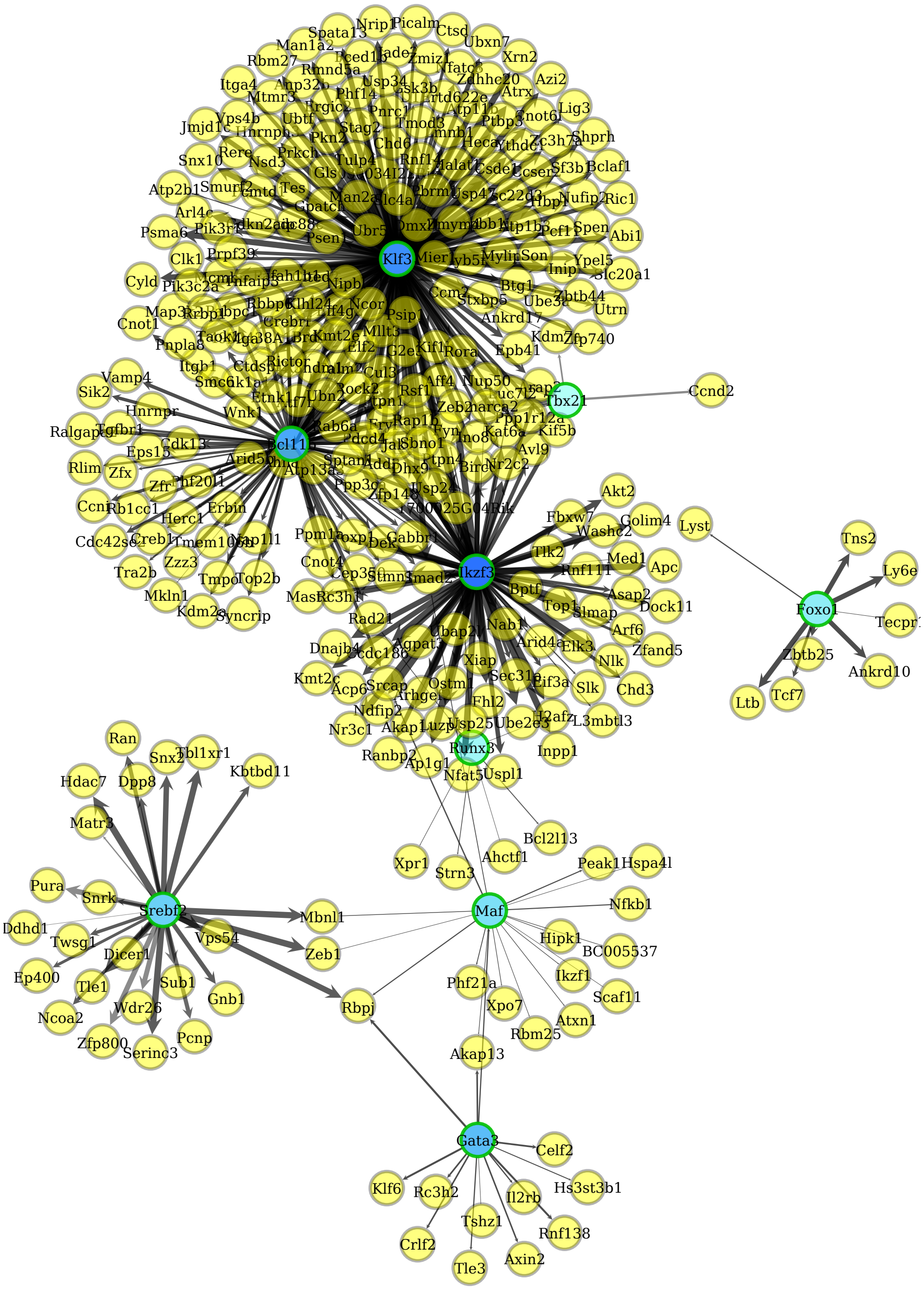

Fig. S6. SwichTFI transition GRN for spleen tissue in the Th1 dataset. TFs are outlined in green. Node fill intensity (blue) indicates TF rank, with darker shades denoting greater importance to the cell state transition, as determined by PageRank centrality in the reversed network. Edge thickness is proportional to the edge weight, and edge opacity is proportional to the empirical *P*-value of the edge, with higher opacity indicating greater statistical significance.

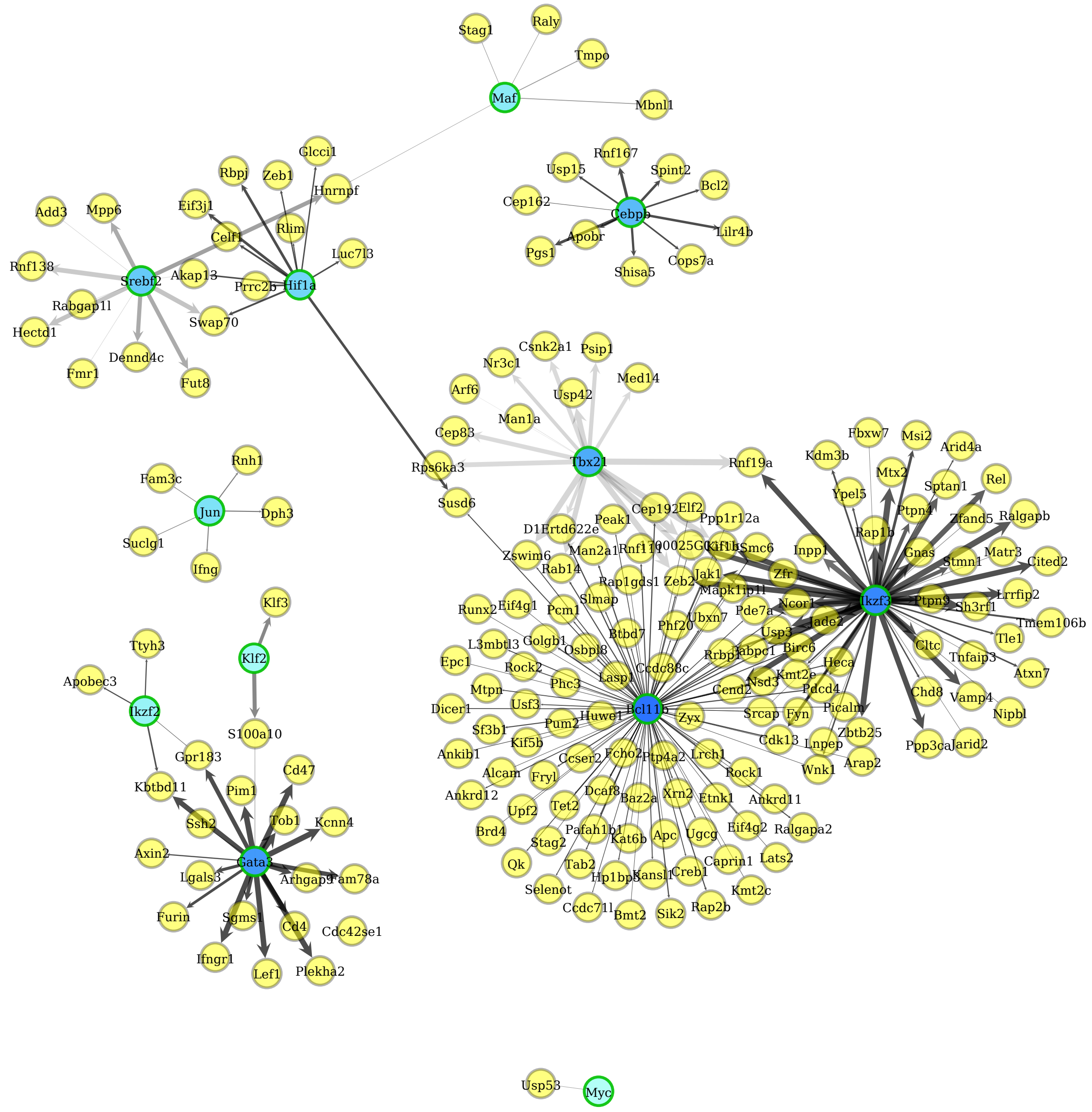

Fig. S7. SwichTFI transition GRN for liver tissue in the Th1 dataset. TFs are outlined in green. Node fill intensity (blue) indicates TF rank, with darker shades denoting greater importance to the cell state transition, as determined by PageRank centrality in the reversed network. Edge thickness is proportional to the edge weight, and edge opacity is proportional to the empirical *P*-value of the edge, with higher opacity indicating greater statistical significance.

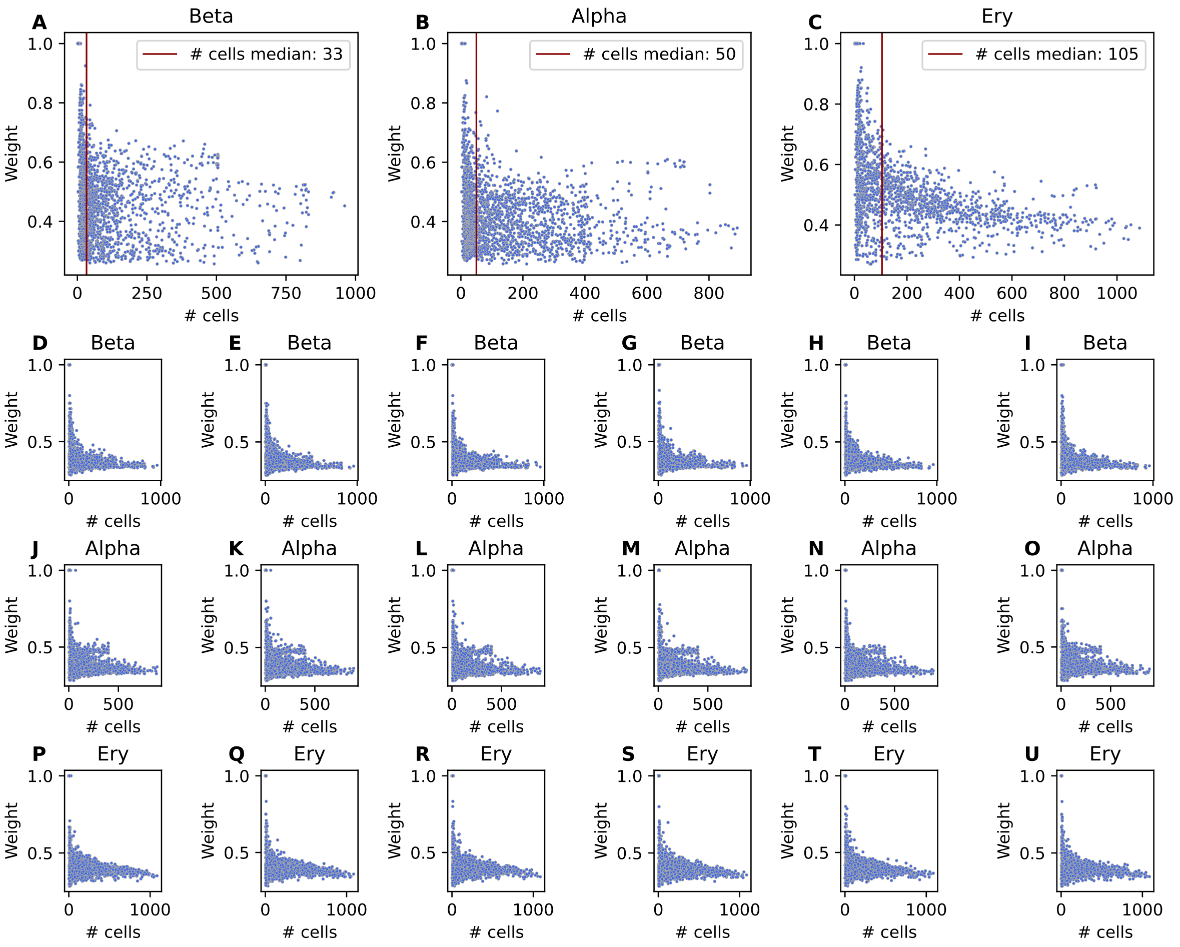

Fig. S8. (A–C) Scatter plots showing the relationships between edge relevance weights and the numbers of cells with TF-target co-expression used for model fitting in the β-cell (A), α-cell (B), and erythrocyte (C) development datasets, obtained when running SwitchTFI without MAGIC imputation. The vertical red lines mark the median numbers of available cells across edges in the baseline GRN. (D–U) Six instances of relationships between numbers of cells available for model fitting weights computed with permuted progenitor-offspring labels (D–I: β-cell data; J–O: α-cell data; P–U: erythrocyte data). The consistent pattern of high weight variances in the low cell count regime across the permuted and non-permuted case indicates that weights for edges with few cells with TF-target co-expression are primarily driven by artifacts due to limited data rather than by true biological signal.

Table S7. Putative driver genes

Dataset	CellRank	spliceJAC	DrivAER	SwitchTFI outdeg	SwitchTFI
Pre-enocr.-β	Ins1, Ins2, Sytl4, Ppp1r1a, Arhgap36, Nnat, Slc2a2, Ero1lb, Calr, Iapp, Gip, G6pc2, Sec24d, Pdia5, Pdia6, Hsp90b1, Papss2, Ttc28, Fkbp2, Itpkb	Pyy, Iapp, Ins2, Rbp4, Ins1, Nnat, Chgb, Malat1, Ttr, Cck, Rpl18a, Pcsk2, Actg1, Dlk1, Chga, Meis2, Runx1t1, Fev, Sec61b, Krt7	Pdx1, Xbp1, Nkx6-1, Pax4, Ybx1, Foxa3, Fos, Etv1, Fosb, Deaf1, Pax6, Fev, Junb, Elk3, Cdx2, Nr3c1, Foxa2, Ilf2, Tbp, Rad21	Xbp1, Pdx1, Pax4, Ybx1, Fev, Etv1, Yy1, Foxa3, Foxa2, Gata6, Jund, Lhx1, Sox4, Nr3c1, Atf3, Mnx1, Elk1, Maff, Zbtb7b, Mxd4	Pdx1, Mnx1, Xbp1, Pax4, Ybx1, Fev, Yy1, Foxa3, Etv1, Lhx1, Nr3c1, Foxa2, Gata6, Elk1, Maff, Sox4, Jund, Atf3, Srebf2, Mxd4
Pre-enocr.-α	Rbp4, Isl1, Pyy, Slc16a10, Peg10, Tmem27, Slc38a5, Irx2, Arg1, Lrpprc, Gast, Tmsb15l, Ank, Meis2, Anpep, Gcg, Scgn, Hap1, Ttr, Iapp	Pyy, Gcg, Iapp, Ttr, Chgb, Rbp4, Meis2, Chga, Cck, Auts2, Rps5, Tmem27, Rps9, Pcsk2, Slc38a5, Fev, Pcsk1n, Krt7, H3f3b, Mdk	Pdx1, Mnx1, Sox9, Arx, Fos, Junb, Etv1, E2f1, Pax4, Klf8, Foxa3, Clock, Jund, Taf6, Maff, Elf2, Zfp467, Foxa2, Xbp1, Fosb	Pax4, Pdx1, Creb3l2, Mnx1, Jund, Arx, Maff, Klf8, Etv1, Foxa3, Foxa2, Jun, Xbp1, Sox9, Klf3, Sox4, Ing4, Gata6, Etv5, Nkx6-1	Pax4, Mnx1, Pdx1, Creb3l2, Jund, Cdx2, Nkx6-1, Arx, Klf8, Maff, Jun, Foxa3, Etv1, Foxa2, Xbp1, Ing4, Etv5, Sox9, Klf3, Rfx3
Erythrocytes	Nusap1, Prc1, Arl6ip1, Top2a, Spire1, Mrpl47, Ckap5, Ube2c, Usp15, Rrm1, Polq, Gnl3, SMC4, Hsph1, Psmb7, Cenpf, Cenpe, Celf1, C530008M17Rik, Jmjd1c	-	Foxo1, Gata1, Mbd2, Etv6, Tfdp2, Irf1, Stat1, Sox4, Arid3a, E2f2, Irf7, Chd1, Dido1, Elk3, E2f8, Ybx1, Bclaf1, Myc, Gata2, Cebpe	Mbd2, Gata2, Myc, Foxo1, Fli1, Gata1, Cux1, Myb, Elk3, Ikzf2, Sox4, Rara, Ybx1, Irf1, Nfe2, Nfia, Tcf3, Ets1, Etv6, Tal1	Mbd2, Foxo1, Gata2, Ybx1, Etv6, Fli1, Gata1, Myc, Cebpa, Sox4, Ikzf2, Cux1, Myb, Elk3, Taf1, Irf1, Nfe2, Pbx1, Rara, Nfia

Table S8. DIGEST results α

Database	Method	DIGEST score	DIGEST p-val
GO.BP	CellRank	0.007578	0.004995
	spliceJAC	0.014933	0.000999
	DrivAER	0.077007	0.000999
	SwitchTFI outdeg	0.077127	0.000999
	SwitchTFI	0.087824	0.000999
GO.CC	CellRank	0.131471	0.000999
	spliceJAC	0.129550	0.000999
	DrivAER	0.365702	0.000999
	SwitchTFI outdeg	0.387706	0.000999
	SwitchTFI	0.376359	0.000999
GO.MF	CellRank	0.115743	0.000999
	spliceJAC	0.121951	0.000999
	DrivAER	0.341738	0.000999
	SwitchTFI outdeg	0.308004	0.000999
	SwitchTFI	0.320063	0.000999
KEGG	CellRank	0.008211	0.001998
	spliceJAC	0.018192	0.000999
	DrivAER	0.039478	0.000999
	SwitchTFI outdeg	0.062736	0.000999
	SwitchTFI	0.062736	0.000999

Table S9. DIGEST results β

Database	Method	DIGEST score	DIGEST p-val
GO.BP	CellRank	0.008555	0.005994
	spliceJAC	0.010089	0.000999
	DrivAER	0.066030	0.000999
	SwitchTFI outdeg	0.071786	0.000999
	SwitchTFI	0.068847	0.000999
GO.CC	CellRank	0.071508	0.000999
	spliceJAC	0.112103	0.000999
	DrivAER	0.302055	0.000999
	SwitchTFI outdeg	0.308665	0.000999
	SwitchTFI	0.315826	0.000999
GO.MF	CellRank	0.067845	0.001998
	spliceJAC	0.108033	0.000999
	DrivAER	0.268302	0.000999
	SwitchTFI outdeg	0.330196	0.000999
	SwitchTFI	0.329223	0.000999
KEGG	CellRank	0.012178	0.002997
	spliceJAC	0.009668	0.000999
	DrivAER	0.048714	0.000999
	SwitchTFI outdeg	0.035819	0.000999
	SwitchTFI	0.035819	0.000999

Table S10. DIGEST results erythrocytes

Database	Method	DIGEST score	DIGEST p-val
GO.BP	CellRank	0.007711	0.006993
	DrivAER	0.060844	0.000999
	SwitchTFI outdeg	0.070138	0.000999
	SwitchTFI	0.056724	0.000999
GO.CC	CellRank	0.113687	0.000999
	DrivAER	0.328629	0.000999
	SwitchTFI outdeg	0.369303	0.000999
	SwitchTFI	0.343111	0.000999
GO.MF	CellRank	0.129266	0.000999
	DrivAER	0.210721	0.000999
	SwitchTFI outdeg	0.258650	0.000999
	SwitchTFI	0.247286	0.000999
KEGG	CellRank	0.000000	1.000000
	DrivAER	0.012989	0.000999
	SwitchTFI outdeg	0.017921	0.000999
	SwitchTFI	0.022444	0.000999

Table S11. Average pairwise Jaccard similarities for input and transition GRNs (rounded to 4 decimals).

Dataset	vertices				edges	
	input	trans.	top 10 TFs (PageRank)	top 10 TFs (outdeg.)	input	trans.
Pre-endocr. α-cell	0.4834	0.2758	0.5606	0.5507	0.1566	0.1817
Pre-endocr. β-cell	0.4956	0.3356	0.5632	0.5634	0.1607	0.2122
Erythrocytes	0.5991	0.3724	0.6994	0.7016	0.2553	0.2147
